# A Novel Gastrodin Derivative with Neuroprotection Promotes NGF-Mimic Activity by Targeting INSR and ACTN4 to Activate PI3K/Akt Signaling Pathway in PC12 Cells

**DOI:** 10.3390/antiox14030344

**Published:** 2025-03-14

**Authors:** Jiayuan Zeng, Jianxia Mo, Makoto Muroi, Hiroyuki Osada, Lan Xiang, Jianhua Qi

**Affiliations:** 1 College of Pharmaceutical Sciences, Zhejiang University, Yu Hang Tang Road 866, Hangzhou 310058, China; 22219069@zju.edu.cn (J.Z.);; 2 Chemical Biology Research Group, RIKEN Center for Sustainable Resource Science, Wako 351-0198, Saitama, Japan

**Keywords:** gastrodin derivatives, nerve growth factor, neuroprotection, insulin receptor, alpha-actinin-4

## Abstract

Gastrodin (gas) has been shown to promote neuroprotection and reverse Alzheimer’s disease (AD) pathology. However, its high effective dose limits its potential in treating AD. In this study, a bioassay system using PC12 cells and the nerve growth factor (NGF)-mimic effect was employed to investigate the structure–activity relationship of gas derivatives. Among the synthesized compounds, GAD037 demonstrated the highest NGF-mimic activity, surpassing gas. Additionally, GAD037 exhibited significant neuroprotective effects, reducing reactive oxygen species (ROS) and malondialdehyde (MDA) levels, thereby improving the survival of PC12 cells under oxidative stress. It also protected cells from A*β*-induced toxicity. Target protein identification and mechanistic studies revealed that insulin receptor (INSR) and alpha-actinin-4 (ACTN4) are potential targets of GAD037, confirmed through specific inhibitors, small interfering RNA (siRNA) analysis, a cellular thermal shift assay (CETSA), and drug affinity responsive target stability (DARTS). Moreover, the phosphatidylinositol 3-kinase (PI3K)/protein kinase B (Akt) and rat sarcoma (Ras)/protooncogene serine–threonine protein kinase (Raf)/mitogen-activated protein kinase (MEK)/extracellular signal-regulated kinase (ERK) signaling pathways were found to be involved in the NGF-mimic activity of GAD037. In conclusion, GAD037 exhibits superior NGF-mimic and neuroprotective activities compared to gas, suggesting its potential as a lead compound for anti-AD applications.

## 1. Introduction

Currently, approximately 50 million individuals suffer from Alzheimer’s disease (AD) and related forms of dementia, with no effective cure available. By 2050, the global prevalence of AD is projected to triple [1]. AD is a neurodegenerative disorder characterized by the pathological accumulation of two types of protein deposits in the brain: amyloid-*β* (A*β*) and tau tangles [2]. Oxidative stress plays a critical role in A*β* toxicity, with reactive oxygen species (ROS) accumulating in A*β* plaque-associated dystrophic neurites observed in the brains of AD patients [3]. Elevated ROS levels can mediate neuronal damage and synaptic dysfunction, ultimately contributing to the progression of AD [4]. Neuroprotective therapies aimed at shielding or reversing brain damage caused by A*β* and oxidative stress are considered ideal strategies for effectively mitigating the development of AD [5].

Nerve growth factor (NGF) is the first recognized neurotrophic factor that enhances neuroprotection, improves learning and memory, and alleviates AD-related pathology [6]. Nevertheless, its therapeutic use in AD patients is limited by poor pharmacokinetics and inadequate blood–brain barrier (BBB) permeability [7]. Consequently, there has been increased interest in the development of small molecules that exhibit NGF-mimic activity as potential treatments for neurodegenerative disorders such as AD [8]. LM11A-31 is the first small molecule regulator targeting the NGF receptor p75^NTR^, demonstrating the potential to mitigate neuron loss induced by A*β* in preclinical models [9]. Small molecules with NGF-mimic activity may offer a novel therapeutic avenue for the management of AD [10].

Numerous anomalies in AD pathogenesis, such as increased A*β* deposition, oxidative stress, and disruptions in energy metabolism, have been shown to result from impaired insulin signaling in the central nervous system [11]. The insulin receptor (INSR) receives insulin signals to activate two key downstream signaling pathways: phosphatidylinositol 3-kinase (PI3K)/protein kinase B (Akt) and rat sarcoma (Ras)/protooncogene serine-threonine protein kinase (Raf)/mitogen-activated protein kinase (MEK)/extracellular signal-regulated kinase (ERK), which are essential for regulating cell metabolism, growth, and differentiation [12]. In the brains of AD patients, a decrease in the expression of insulin-signaling-related proteins has been observed, suggesting a link between impaired insulin signaling and the progression of the disease [13]. Activation of the PI3K/Akt pathway has been beneficial in promoting the proliferation, survival, and phagocytosis of microglia, thereby protecting neurons from A*β*-induced neurotoxicity and improving cognitive impairment [14]. Additionally, overexpression of alpha-actinin-4 (ACTN4) has been found to alleviate memory impairment and reverse chronic neuropathic pain-related changes in dendritic spines [15]. Thus, to elucidate the mechanism of action of GAD037, this study focuses on these signaling pathways.

*Gastrodia elata* Blume, a well-known traditional Chinese medicine (TCM), is primarily used to treat nervous system disorders such as headaches, epilepsy, and stroke [16]. Gas, the main bioactive component of *Gastrodia elata* Blume, was approved for the treatment of sedation and neurasthenia in 1984 [17]. Recent studies have shown that gas may protect hippocampal and cortical neurons from A*β*-induced neurotoxicity, potentially reversing memory deficits and reducing oxidative stress [18]. In earlier research, we found that gas can exert anti-AD effects through neuroprotective mechanisms [19]. However, its effectiveness is suboptimal, with an effective dose established in mice at a minimum of 90 mg/kg. Therefore, there is a pressing need to develop a lead compound with enhanced anti-AD efficacy.

GAD037 is an active lead compound which was obtained through a structure–activity relationship study based on the chemical structure of gas. The NGF-mimic effect of GAD037 is a twofold of gas and no toxicity at a concentration of 200 µM. Therefore, we selected this molecule to conduct an intensive study. Here, we report that GAD037 targets INSR and ACTN4 proteins and regulates downstream PI3K/Akt and Ras/Raf/MEK/ERK signaling pathways to produce the NGF-mimic effect in PC12 cells.

## 2. Materials and Methods

### 2.1. General, Cell Line, Culture Medium

Silica gel (200–300 mesh, Yantai Research Institute of Chemical Industry, Yantai, China) and reversed-phase C18 (octadecylsilyl, ODS) silica gel (Cosmosil 75 C18-OPN, Nacalai Tesque, Kyoto, Japan) were used for column chromatography. Precoated silica gel (0.25 mm) and RP-18 plate (0.25 mm) (Merck KGaA, Darmstadt, Germany) were used for Thin-Layer Chromatography (TLC). Nuclear Magnetic Resonance Spectroscopy (NMR) spectra were recorded on a Bruker AV III-500 spectrometer (Bruker, Karlsruhe, Germany). High-resolution (HR) ESI-TOF-MS were recorded on an Agilent 6224A LC/MS (Agilent Technologies Inc., Santa Clara, CA, USA). Gas was purchased from J&K Scientific Company, Beijing, China.

PC12 cells were purchased from the Cell Bank of the Chinese Academy of Sciences (Shanghai, China) and cultured in Dulbecco’s modified Eagle’s medium (DMEM) (CellMax, Lanzhou, China), supplemented with 10% Horse Serum (HS) (Solarbio, Beijing, China), 7.5% fetal bovine serum (FBS) (CellMax, Lanzhou, China), and 1% Penicillin/Streptomycin (Solarbio, Beijing, China) in a humidified incubator at 37 °C and 5% CO_2_.

### 2.2. Reagents and Antibodies

The following are the sources of the chemicals used in experiments: Dimethyl sulfoxide (DMSO) (CAT No.: D8418), NGF (CAT No.: N2513), tyrosine kinase A (TrkA) inhibitor (k252a, CAT No.: 420298-M), MEK inhibitor (U0126, CAT No.: 19-147), PI3K inhibitor (LY294002, CAT No.: 440202), and glucocorticoid receptor (GR) inhibitor (RU486, CAS No.:84371-65-3) were bought from Sigma-Aldrich Co, Boston, MA, USA. The INSR inhibitor (HNMPA-(AM)_3_, CAT No.: sc-221730), insulin-like growth factor-1 receptor (IGF-1R) inhibitor (T9576, CAS No.: 477-47-4), and Raf inhibitor (AZ628, CAT No.: sc-364418) were obtained from Santa Cruz Biotechnology, Dallas, TX, USA. The Ras inhibitor (FTA, Item No.: 17474) was purchased from Cayman Chemical, Ann Arbor, MI, USA. The pronase E (CAT No.: HY-114158) was bought from MedChemExpress, Shanghai, China, while tyrosine kinase B (TrkB) inhibitor (ANA-12, CAT No.: S7745) was acquired from Selleck, Shanghai, China. Methylthiazolyldiphenyl-tetrazolium bromide (MTT) was purchased from Richu BioScience Co., Ltd., Shanghai, China.

The antibodies against INSR (CAT No.: 3025S), phospho-INSR (Tyr1146, CAT No.: 3021T), PI3K (CAT No.: 4249S), phospho-PI3K (Tyr458, CAT No.: 4228S), Akt (CAT No.: 9272S), phospho-Akt (Ser473, CAT No.: 9271S), ERK (CAT No.: 12041T), and phospho-ERK (CAT No.: 2261S) were acquired from Cell Signaling Technology, Boston, MA, USA. The β-actin antibody (CAT No.: CW0096M), secondary antibodies horseradish peroxidase-linked anti-mouse (CAT No.: CW0102S) and anti-rabbit IgGs (CAT No.: CW0103S), and the pico-ECL Western blot chemiluminescence detection kit (CAT No.: CW0049M) were obtained from Beijing CoWin Biotech Company, Beijing, China.

### 2.3. Synthesis of Gastrodin Derivative GAD037

The synthesis route of GAD037 is illustrated in Figure S1, with the synthesis process comprising four steps. Initially, compound **1** was synthesized by the borane reduction of 2,6-difluoro-4-hydroxybenzonitrile, followed by Boc protection. Subsequently, 2,3,4,6-Tetra-*O*-acetyl-α-*D*-glucopyranosyl bromide was reacted with compound **1** under basic conditions to yield compound **2**. After deacetylation in saturated methanol containing NaHCO_3_ and ethoxylation in the presence of DEPC, compound **3** was obtained. Finally, the glycosyl group underwent acetylation with acetic anhydride in the presence of catalytic pyridine, resulting in the formation of GAD037. Additional details are provided in the Supporting Information.

The chemical structure of the GAD037 was identified by HR ESI-MS, ^1^H NMR, and ^13^C NMR spectra as shown in Figure S2. Spectra data were as follows: HR ESI-TOF-MS *m*/*z* 642.1975, calcd. for C_27_H_35_F_2_NO_13_Na [M+Na]^+^ 642.1969. ^1^H NMR (500 MHz, CDCl_3_): *δ* (ppm) = 6.55–6.52 (2H, m), 5.29 (1H, m), 5.23 (1H, m), 5.11 (1H, m), 5.03 (1H, d, *J* = 7.6 Hz), 4.32–4.28 (3H, m), 4.22–4.15 (3H, m), 3.90 (1H, m), 2.07 (3H, s), 2.06 (3H, s), 2.03 (3H, s), 1.43 (9H, s), 1.30 (3H, t, *J* = 7.1 Hz). ^13^C NMR (125 MHz, CDCl_3_): *δ* (ppm) = 170.17, 169.48, 169.17, 161.76 (dd, ^1^*J* (C, F) = 248.4, ^3^*J* (C, F) = 11.1 Hz), 157.09 (t, ^3^*J* (C, F) = 14.2 Hz), 155.35, 154.74, 109.24, 100.88 (dd, ^2^*J* (C, F) = 29.6 Hz, ^4^*J* (C, F) = 7.2 Hz), 98.71, 79.64, 72.43, 72.23, 70.91, 68.34, 65.28, 64.55, 32.20, 28.34, 20.56, 14.12.

### 2.4. Evaluation of NGF-Mimic Activity in PC12 Cells

Our previous study comprehensively described the assessment of the NGF-mimic activity in PC12 cells [20]. In a 24-well microplate, approximately 5 × 10^4^ cells per well were cultured under appropriate conditions for 24 h. The medium in each well was replaced with 1 mL of DMEM, containing either the test samples dissolved in DMSO or 0.5% DMSO alone. The positive control consisted of NGF at a concentration of 40 ng/mL, and 0.5% DMSO served as the negative control. After a 48 h culture period, around 100–200 cells were randomly selected and counted three times. Positive cells were defined as those exhibiting neurite outgrowth longer than the diameter of their cell bodies. The NGF-mimic activity of PC12 cells treated with the various compounds was expressed as the proportion of positive cells within the selected area.

In the inhibitor experiment, cell culture and seeding proceeded as previously described. Cells were pretreated for 30 min with 500 µL of DMEM containing the specific inhibitors. Following this, the medium in each well was supplemented with 500 µL of DMEM containing the test samples dissolved in DMSO or 0.5% DMSO alone. After a 48 h incubation, the NGF-mimic activity was evaluated as described above.

### 2.5. Cell Viability Assay

An MTT assay was conducted to assess the cell viability following methods outlined in previous studies [21]. In general, cell culture and seeding of cells were performed as previously stated. Cells were subsequently incubated with GAD037 or gas at concentrations of 0, 0.1, 1, and 10 µM, with 0.5%DMSO serving as the negative control. After 24 h, 500 µL of DMEM containing MTT (0.2 mg/mL) was added to each well, replacing the original medium, and the plates were incubated for an additional 2 h. Absorbance was measured at 570 nm using a plate reader (BioTek Synergy H1, Agilent, Santa Clara, CA, USA).

### 2.6. Neuroprotection Assay of GAD037 on PC12 Cells

Initially, we established a hydrogen peroxide (H_2_O_2_) damage model, selecting a 1 h treatment with 0.8 mM H_2_O_2_ for 1 h as the optimal condition. Approximately 5 × 10^4^ cells per well were seeded in a 24-well plate and cultured for 24 h. The medium in each well was replaced with 1 mL of DMEM containing the test compound dissolved in DMSO or 0.5% DMSO alone and cultured for 24 h. Following this, the medium was removed and the cells were exposed to H_2_O_2_ (0.8 mM) for 1 h. Cell viability was then assessed using the MTT assay.

In the construction of the A*β* damage model, we investigated the optimal treatment duration and concentration of A*β*_25–35_, identifying 30 µM A*β*_25–35_ for 48 h as the most effective condition. Subsequently, approximately 5 × 10^3^ PC12 cells were seeded into 96-well plates and allowed to incubate for 24 h. The medium in each well was then replaced with DMEM containing the test compound dissolved in DMSO or 0.5% DMSO alone and incubated for 30 min, before adding A*β*_25–35_ at a final concentration of 30 µM. After incubation in a cell culture environment for 48 h, the cell viability was assessed using the MTT assay in the 96-well plates.

### 2.7. RNA Interference

The primer sequences used for generating knockdown of INSR and ACTN4, along with the negative control (NC) siRNA, are detailed below. For INSR (NM_017071.2), sense: 5′-GUG AAG AGC UGG AGA UGG ATT-3′; anti-sense: 5′-UCC AUC UCC AGC UCU UCA CTT-3′. For ACTN4 (NM_031675.2), sense: 5′-UUG AAG UGG CUG AGA AAU ATT-3′; anti-sense: 5′-UAU UUC UCA GCC ACU UCA ATT-3′. For NC siRNA, sense: 5′-UUC UCC GAA CGU GUC ACG UTT-3′; anti-sense: 5′-ACG UGA CAC GUU CGG AGA ATT-3′. In line with the manufacturer’s instructions, PC12 cells were transfected with FAM-labeled siRNA at concentrations of 50, 100, and 150 nM, respectively, to assess the transfection efficacy. The optimal final concentration of 150 nM for either INSR siRNA or ACTN4 siRNA was chosen to achieve a transfection efficiency exceeding 90% (Figure S3). Approximately 5 × 10^4^ cells were then plated in each well of 24-well plates and cultured in an antibiotic-free growth medium for 24 h. Subsequently, 0.5 mL of DMEM containing 1 µL of Lipofectamine RNAiMAX (Thermo Fisher Scientific, Waltham, MA, USA), was combined with 0.5 mL of DMEM containing either INSR siRNA, ACTN4 siRNA, or NC siRNA at a final concentration of 150 nM, and this mixture replaced the original medium. After an 8 h transfection period, the medium in each well changed to 1 mL of DMEM containing GAD037, a concentration of 10 µM dissolved in DMSO, or 0.5% DMSO alone for an additional 48 h culture. Changes in NGF-mimic activity were then evaluated. For the assessment of INSR and ACTN4 protein levels following transfection with different siRNAs, approximately 2 × 10^6^ cells were plated in each 6 cm cell culture dish. The concentrations of siRNA, transfection reagent, and transfection duration remained consistent with the earlier protocols. Cell proteins were extracted from each culture dish after treatment with GAD037 or 0.5% DMSO for 2 h and analyzed using Western blotting.

### 2.8. Determination of ROS and MDA Levels

To determine the levels of ROS in PC12 cells, approximately 5 × 10^4^ PC12 cells were seeded in each well of a 24-well plate. The cells were incubated with gas (10 µM) or GAD037 (0.1, 1, and 10 µM) for 24 h, followed by exposure to 0.8 mM H_2_O_2_ for 1 h. 2,7-dichlorodihydrofluorescein diacetate (DCFH-DA) (Beyotime Biotechnology Inc., Shanghai, China) was added to each well at a final concentration of 10 µM and incubated for 30 min. The cells were then washed with phosphate-buffered saline (PBS) to remove any extracellular DCFH-DA, and intracellular ROS levels in the PC12 cells were assessed using a fluorescence microscope (HCS, Thermo Fisher, Science, Waltham, MA, USA). For the measurement of ROS levels in PC12 cells transfected with different siRNAs, the culture and seeding of the cells were performed as described above. Lipofectamine RNAiMAX was then mixed with siRNA (INSR siRNA, ACTN4 siRNA, or NC siRNA) at a final concentration of 150 nM in DMEM. The mixture replaced the original medium. Following an 8 h transfection period, the concentration and treatment time of the compound, as well as the detection of ROS levels, were conducted as previously detailed.

Malondialdehyde (MDA) quantification was carried out using MDA assay kits (Nanjing Jiancheng Bioengineering Institute, Nanjing, China) in accordance with the manufacturer’s instructions. Initially, approximately 2 × 10^6^ PC12 cells were seeded in each 60 mm dish and cultured for 24 h. The PC12 cells were then treated with gas (10 µM) or GAD037 (0.1, 1, and 10 µM) for another 24 h, followed by exposure to 0.8 mM H_2_O_2_ for 1 h. After this, the cells were lysed and centrifuged at 12,000 rpm for 10 min at 4 °C. The protein from each sample was collected for MDA level detection. To measure MDA levels in PC12 cells transfected with different siRNAs, the culture and seeding of the cells were conducted as described previously. Subsequently, the medium in each dish was replaced with a mixture of Lipofectamine RNAiMAX and siRNA (INSR siRNA, ACTN4 siRNA, or NC siRNA) at a final concentration of 150 nM and incubated for 8 h. The concentration and treatment time of the compound, along with the evaluation of MDA levels, were performed as stated above.

### 2.9. Western Blot Analysis

Western blot analysis was performed according to the procedures outlined in previous studies [21]. To extract proteins, cells were homogenized in RIPA lysis buffer supplemented with 1% protease and 1% phosphatase inhibitors. The samples were then centrifuged at 12,000 rpm at 4 °C for 10 min and the supernatant was transferred to new tubes. Protein concentrations were determined using a BCA kit (CoWin Biotech Co., Ltd., Taizhou, Jiangsu, China), and all samples were denatured for 10 min at 100 °C. Approximately 20 µg of protein from each sample was loaded into each well of the sodium dodecyl sulfate-polyacrylamide gel (SDS-PAGE) gel. Gel electrophoresis was conducted at 80 V for 15 min, followed by 120 V for 60 min. Following electrophoresis, the proteins were transferred to a polyvinylidene fluoride (PVDF) (Bio-Rad Laboratories Inc., Hercules, CA, USA) membrane for 90 min, after which they were blocked with 5% skimmed milk for 60 min. The membrane was then incubated with a diluted primary antibody at 4 °C overnight. After washing, the membrane was incubated with a secondary antibody for 45 min. Protein bands were then visualized using a chemiluminescence detection kit (Beijing Cowin Biotech Company, Beijing, China), and blot density was quantified using ImageJ software (Version 1.42q, National Institutes of Health, Rockville, MD, USA). The specific primary and secondary antibodies utilized in this study are detailed in the antibodies section of the Materials and Methods.

### 2.10. Thermal Shift Assay (CETSA)

CETSA is an effective target validation technique, executed according to previously reported procedures [20]. Initially, approximately 2 × 10^6^ cells were inoculated into each 6 cm cell culture dish and cultured for 24 h. The medium was subsequently replaced with 5 mL of serum-free DMEM medium containing GAD037 at a concentration of 10 µM dissolved in DMSO or 0.5% DMSO alone, followed by an additional 4 h incubation. Proteins from each group were then extracted, and the protein concentration was measured using a BCA kit. Both the control and treatment groups were divided into seven equal aliquots. Each aliquot, containing 100 µL of protein (2 µg/µL), was heated at temperatures of 46, 50, 54, 58, 62, and 66 °C for 3 min. The supernatant from each sample was collected by centrifugation at 12,000 rpm at 4 °C for 20 min. Each sample was then mixed with 5 × SDS-PAGE loading buffer, denatured at 100 °C for 10 min, and analyzed by Western blotting.

### 2.11. Drug Affinity Responsive Target Stability (DARTS)

The DARTS procedure was performed as previously described [20]. The PC12 cell proteins were harvested, and their concentrations were measured using the BCA kit assay, subsequently diluting the samples of 2 µg/µL. The protein samples were divided into two equal aliquots and incubated with either 0.5% DMSO or GAD037 at a concentration of 10 µM for 3 h at room temperature. Following this incubation, both the control and GAD037 treatment groups were further divided into five equal aliquots. Each aliquot was treated with pronase E (MedChemExpress, Shanghai, China) at ratio of 500:1, 200:1, 100:1, 50:1 (protein:pronase = *w*/*w*) or an equivalent volume of TNC buffer (50 mM Tris-HCl, 50 mM NaCl, 10 mM CaCl_2_) at 25 °C for 25 min. After treatment, all samples were mixed with 5 × SDS-PAGE loading buffer and heated at 100 °C for 10 min in preparation for Western blotting. For the concentration–response DARTS experiment, the protein samples were divided into seven equal aliquots, which were incubated with 0.5% DMSO or GAD037 at concentrations of 0.1, 1, 5, 10, or 25 µM for 3 h at room temperature. Each sample underwent treatment with protease E (protein:pronase = 100:1 *w*/*w*) or an equivalent volume of TNC buffer at 25 °C for 25 min, with subsequent processing following the aforementioned protocol.

### 2.12. 2D Gel Electrophoresis-Based Proteome-Wide CETSA (2DE-CETSA)

The 2DE-CETSA procedure was carried out according to previously established methods [22]. In brief, the HeLa cell line was obtained from RIKEN Cell Bank (RIKEN BRC, Tsukuba, Japan). The cells were cultured in DMEM (Thermo Fisher Scientific, Waltham, MA, USA) supplemented with 10% fetal bovine serum (Sigma-Aldrich, St. Louis, MO, USA), 50 units/mL penicillin (Thermo Fisher Scientific, Waltham, MA, USA), and 50 μg/mL streptomycin (Thermo Fisher Scientific, Waltham, MA, USA) at 37 °C in a humidified incubator under 5% CO_2_ for five days after resuscitation. When the cells reached 80% coverage, the cells were then washed thrice with PBS, lysed with RIPA lysis buffer containing 1% protease inhibitor cocktail, and incubated on ice for 20 min. The cell lysates were centrifuged and the supernatant was removed for the cellular thermal shift assay. In brief, HeLa cell lysates (2 µg/µL protein) were treated with either gas (50 µM) or DMSO and heated at 40, 45, 50, 55, and 60 °C for 5 min. Proteins from each sample were collected via centrifugation, labeled with various fluorescent dyes (Cy2 or Cy5), and subsequently analyzed using 2-D DIGE. The log ratios of protein abundance for samples treated with gas compared to DMSO at each spot were examined by generating melting curves. Subsequently, selected spots displaying shifts in thermal stability were identified through LC-MS/MS analysis.

### 2.13. Quantification and Statistical Analysis

Statistical analysis was performed by using GraphPad Prism 8.0 (GraphPad Software, San Diego, CA, USA). The statistical significance of differences between two experimental groups was assessed using an unpaired *t*-test, while differences between three or more groups with normally distributed values were evaluated using one-way ANOVA. Data are presented as mean ± SEM, and statistical significance was defined as *p* < 0.05.

## 3. Results

### 3.1. GAD037 Exerts NGF-Mimic Activity in PC12 Cells

**Figure 1:**
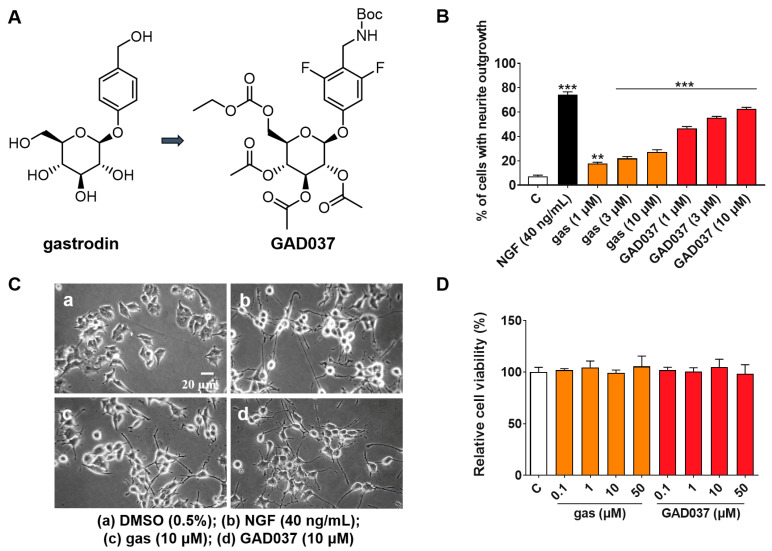
Effects of gas and GAD037 on neurite outgrowth and cell viability in PC12 cells. (**A**) Chemical structures of gas and GAD037. (**B**) Percentage of PC12 cells with neurite outgrowth following treatment with gas or GAD037 at concentrations of 1, 3, and 10 µM for 48 h. Positive control consisted of NGF at concentration of 40 ng/mL, and 0.5% DMSO served as negative control. (**C**) Morphological changes of neurite outgrowth in PC12 cells. (**D**) Cell viability following exposure to gas or GAD037 at concentrations of 0.1, 1, 10, and 50 µM for 24 h. Three biologically independent replicates were performed in 3rd, 4th, and 9th generations of cells, and data are presented as mean ± SEM. ** *p* < 0.01 and *** *p* < 0.001 represent significant differences compared with control group.

Gas is a potential anti-AD compound, but its efficacy is suboptimal [19]. To develop novel compounds with enhanced anti-AD properties, we initiated a structure–activity relationship study centered on gas. Among the synthesized derivatives, GAD037 demonstrated the most significant NGF-mimic activity, and its chemical structure was identified (Figure 1A). The synthetic route is shown in Figure S1. Furthermore, GAD037 served as a lead compound for a concentration–response investigation of NGF-mimic activity. As illustrated in Figure 1B,C, the NGF-mimic activity of GAD037 was found to be concentration-dependent. Specifically, 62.7% ± 1.2% of PC12 cells exhibited neurite growth when treated with GAD037 at a concentration of 10 μM, compared to 22.3% ± 1.8% for those treated with gas. These findings indicate that the introduction of fluorine atoms, modification of the 4-substituent on the benzene ring, and alterations to the glycosyl moiety influence the NGF-mimic activity in PC12 cells.

Furthermore, to determine whether GAD037 induces cytotoxicity at effective concentrations of NGF activity, the survival of PC12 cells treated with GAD037 was evaluated. After treatment with GAD037 at concentrations of 0.1, 1, 10, 50, 100, and 200 µM, the viability of PC12 cells was not significantly different from that of the control group (Figure 1D and Figure S4). These results demonstrate that GAD037 exhibits excellent NGF-mimic activity and no cytotoxicity.

### 3.2. GAD037 Exhibits Neuroprotective Activity Through Antioxidative Stress and Anti-Aβ Damage

**Figure 2:**
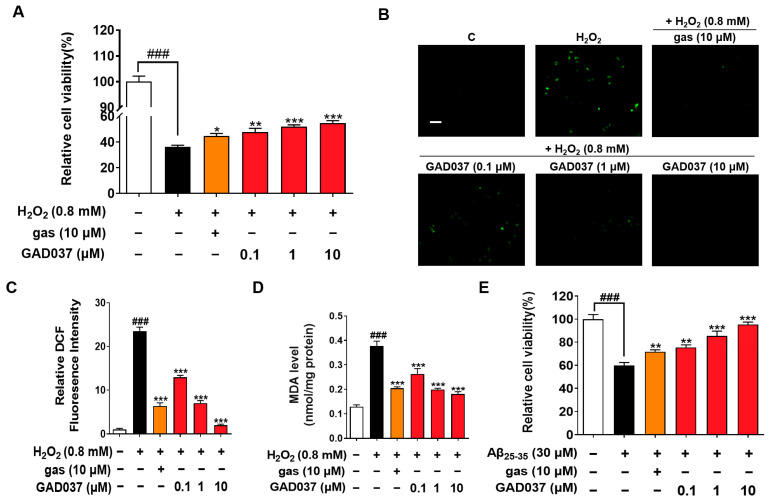
Neuroprotection activity of GAD037 through antioxidative stress and anti-A*β* damage in PC12 cells. (**A**) Cell viability of H_2_O_2_-induced PC12 cells after being treated with GAD037 or gas for 24 h. (**B**) Microphotograph and (**C**) fluorescence intensity quantification of PC12 cells stained with DCFH-DA to detect ROS under fluorescence microscopy. Scale bar, 100 µm. (**D**) Levels of MDA content after treatment with GAD037 or gas in PC12 cells for 24 h. (**E**) Cell viability of A*β*-induced PC12 cells after being treated with GAD037 or gas for 48 h. The white, black, orange, red columns represent the control, the H_2_O_2_ or A*β*_25–35_, H_2_O_2_ or A*β*_25–35_ + gas (10 µM), H_2_O_2_ or A*β*_25–35_ + GAD037 groups, respectively. In evaluation of cytotoxicity, H_2_O_2_ or A*β* damage assay, and ROS assay, 24-well plates were used in experimental design, with three repeat wells in each group. For determination of MDA, experimental design was repeated with three 6 cm dishes in each group, and protein was extracted respectively for determination. Data are presented as mean ± SEM. ^###^ *p* < 0.001 represents significant differences compared with control group; * *p* < 0.05, ** *p* < 0.01, and *** *p* < 0.001 represent significant differences compared with H_2_O_2_ or A*β*_25–35_ group.

Oxidative stress is observed in AD patients, and the brain is particularly vulnerable to oxidative stress, leading to injury and functional impairments. Compounds with antioxidant properties are being explored as potential treatments for neurodegenerative diseases [23]. Previous studies have shown that gas exerts neuroprotective effects in AD-related pathology [18]. To understand whether GAD037 has neuroprotection, the antioxidative stress and anti-A*β* function were evaluated. GAD037 and gas improved H_2_O_2_-induced morphological changes of PC12 cells, including damaged cellular membranes and cellular swelling (Figure S5A). At the same time, it significantly increased the survival rate of PC12 cells exposed to H_2_O_2_ in a concentration-dependent manner. The antioxidant capacity of this molecule at a concentration of 10 µM was higher than that of gas (Figure 2A). Furthermore, ROS and MDA, two key biomarkers of oxidative stress, were measured. GAD037 significantly reduced the H_2_O_2_-induced increase in ROS and MDA in PC12 cells (Figure 2B–D). Moreover, neurotoxicity caused by A*β* can trigger oxidative stress, resulting in neuronal damage or loss [24]. GAD037 remarkably improved the viability and reduced the abnormal shapes of PC12 cells treated A*β* at 30 µM (Figure 2E and Figure S5B). These results suggest that GAD037 exhibits significant neuroprotective activity by alleviating oxidative stress and A*β*-induced damage. GAD037 exhibits superior antioxidant activity compared to gas.

### 3.3. Specific Inhibitors Screen GAD037-Induced NGF-Mimic-Activity-Related Potential Targets and Signaling Pathways

**Figure 3:**
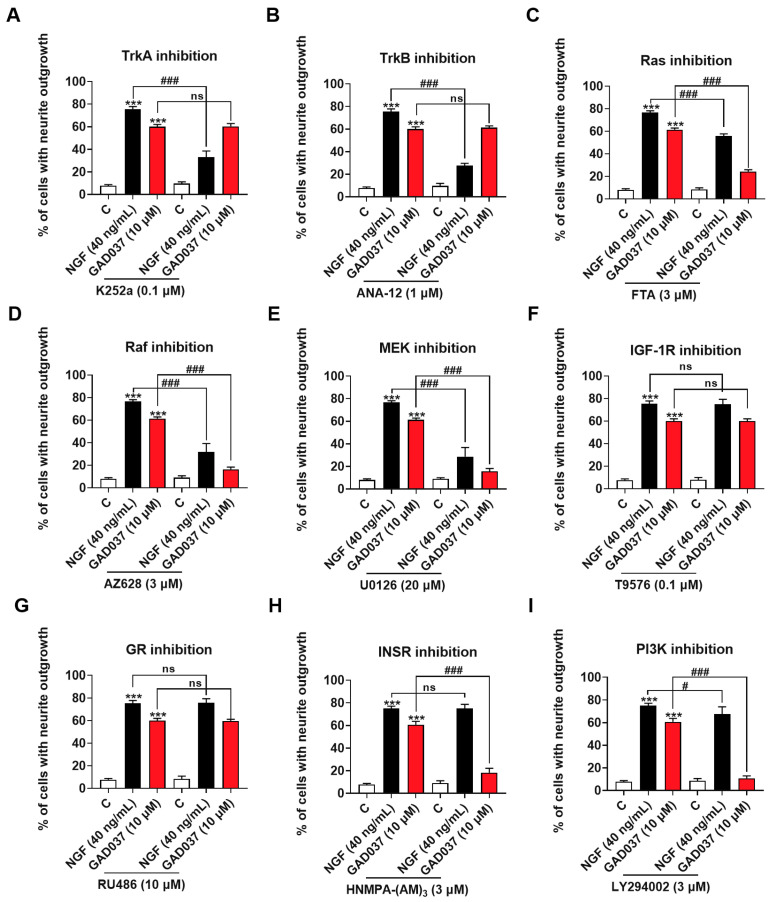
Screening NGF-mimic activity of GAD037-related potential targets and signaling pathways. (**A**–**I**) Effects of inhibitors of TrkA, TrkB, Ras, Raf, ERK, GR, IGF-1R, INSR, PI3K (K252a, ANA-12, FTA, AZ628, U0126, RU486, T9576, HNMPA-(AM)_3_, and LY294002, respectively) on neurite outgrowth induced by GAD037 treatment for 48 h. All experiments with inhibitors were conducted simultaneously in 3rd, 4th, and 9th generations of cells. Data are presented as mean ± SEM. *** *p* < 0.001 represents significant differences compared with control group; ns indicate no significant differences; ^#^ and ^###^ indicate significant differences at *p* < 0.05 and *p* < 0.001, respectively.

To screen candidate target proteins of GAD037, a specific inhibitor experiment was conducted. Numerous signaling pathways associated with promoting neurite outgrowth promotion have been reported. Neurotrophic factors, such as NGF and brain-derived neurotrophic factor (BDNF), have been shown to interact specifically with the tyrosine kinase receptors TrkA and TrkB, activating downstream kinases that facilitate neurite outgrowth, differentiation, and cell survival [8]. Neurotrophin signaling is mediated by several pathways [25], including the Ras/Raf/MEK/ERK and PI3K/Akt pathways driven by tyrosine kinases. Furthermore, phosphorylation of IGF-1R and INSR has been demonstrated to trigger downstream signaling cascades that may modulate cell metabolism and promote neurite outgrowth [13,26]. It has also been suggested that GR is involved in the neurogenic activity of PC12 cells [20]. Consequently, we evaluated whether the treatment with inhibitors of these proteins influenced the NGF-mimic activity induced by GAD037. As illustrated in Figure 3A,B, the NGF-mimic activity of GAD037 remained unchanged following treatment with TrkA and TrkB inhibitors, which indicates that these receptors are not involved in the NGF-mimic activity of GAD037 in PC12 cells. Additionally, a significant reduction in the number of cells exhibiting neurite outgrowth was observed with the treatment with inhibitors targeting Ras, Raf, and ERK (FTA, AZ628, and U0126). As shown in Figure 3C–E, the percentage of neurite outgrowth decreased from 61.3% ± 0.9% to 24.3% ± 0.9%, 16.3% ± 1.2%, and 15.7% ± 1.5%, respectively. These results suggest that the Ras/Raf/MEK signaling pathway may play a critical role in the NGF-mimic activity of GAD037, independent of Trk receptors. Furthermore, as shown in Figure 3F,G, treatment with inhibitors of GR and IGF-1R (RU486 and T9576) did not markedly alter the neurite outgrowth induced by GAD037. Notably, after treatment with INSR and PI3K inhibitors (HNMPA-(AM)_3_ and LY294002) GAD037-induced neurite outgrowth was significantly reduced from 60.8% ± 1.5% to 18.3% ± 1.9% (Figure 3H), and 10.8% ± 1.1% (Figure 3I). These findings indicate that INSR is a candidate target of GAD037, and that the Ras/Raf/MEK and PI3K/Akt signaling pathways contribute to the NGF-mimic activity of GAD037.

### 3.4. GAD037 Targets INSR in PC12 Cells

**Figure 4:**
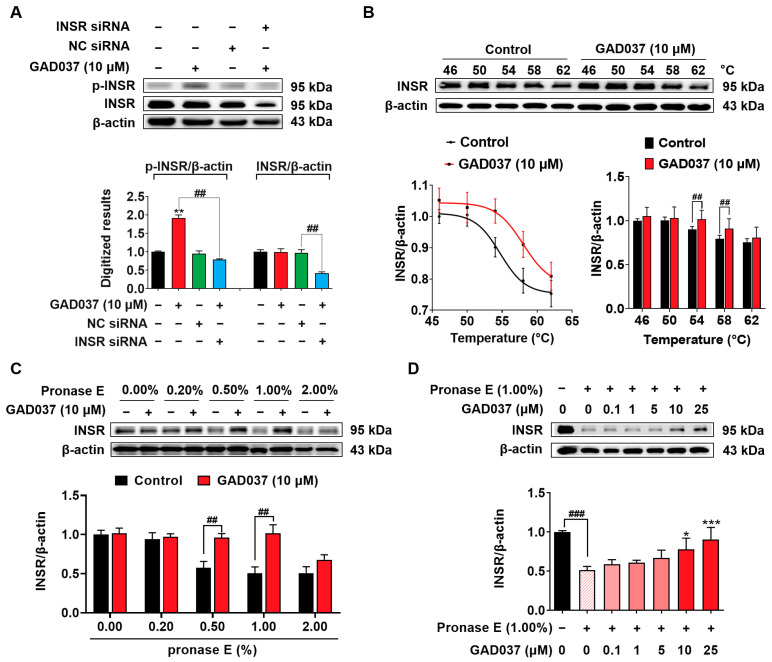
Identification of INSR as one of potential targets of GAD037. (**A**) Western blot analysis and digitalized results of INSR protein and phosphorylated INSR protein following transfection with either INSR siRNA or NC siRNA, and GAD037 treatment for 2 h. (**B**) Western blot analysis and digitalized results of INSR protein after being treated with GAD037 at 10 µM for 4 h and heating samples at different temperatures, respectively. (**C**) PC12 cell lysates and GAD037 at 10 µM were incubated for 3 h at room temperature and digested with pronase E of different concentrations for 25 min, and INSR protein in lysates was determined by Western blot analysis. (**D**) PC12 cell lysates and different concentrations of GAD037 were incubated for 3 h at room temperature and digested with pronase E (0 or 1:100 dilution) for 25 min, and INSR protein in lysates was determined by Western blot analysis. In (**A**–**C**), the black, red, green, blue columns represent the control, GAD037, NC siRNA, INSR siRNA + GAD037 groups, respectively. In (**D**), the black, light pink, pink, red represent the control, pronase E, pronase E + GAD037 (0.1, 1, 5 µM), pronase E + GAD037 (10, 25 µM) groups, respectively. For Western blot analysis, three 6 cm dishes were repeated for each group, and extracted proteins were mixed and sampled. Data are presented as mean ± SEM. * *p* < 0.05, ** *p* < 0.01, and *** *p* < 0.001 represent significant differences compared with control group; ^##^ and ^###^ indicate significant differences at *p* < 0.01 and *p* < 0.001, respectively.

To get more evidence to support that INSR is a target of GAD037, the siRNA of INSR, CETSA, and DARTS analysis were performed. As displayed in Figure 4A, the INSR protein level was dramatically decreased after transfection with INSR siRNA. Additionally, INSR siRNA significantly reduced the phosphorylated INSR protein induced by GAD037. In CETSA, GAD037 enhanced the thermal stability of the INSR protein at the pointed temperature region in Figure 4B. Furthermore, GAD037 at a concentration of 10 μM notably inhibited the degradation of INSR proteins after the treatment with pronase in varying concentrations in the DARTS analysis, especially at 1.0% (Figure 4C). Meanwhile, GAD037 at concentrations of 5, 10, and 25 µM improved the stability of the INSR protein in a concentration-dependent manner (Figure 4D). These findings indicate that INSR is a target protein of GAD037 that produces the NGF-mimic effect.

### 3.5. ACTN4 Is a Target of GAD037

**Figure 5:**
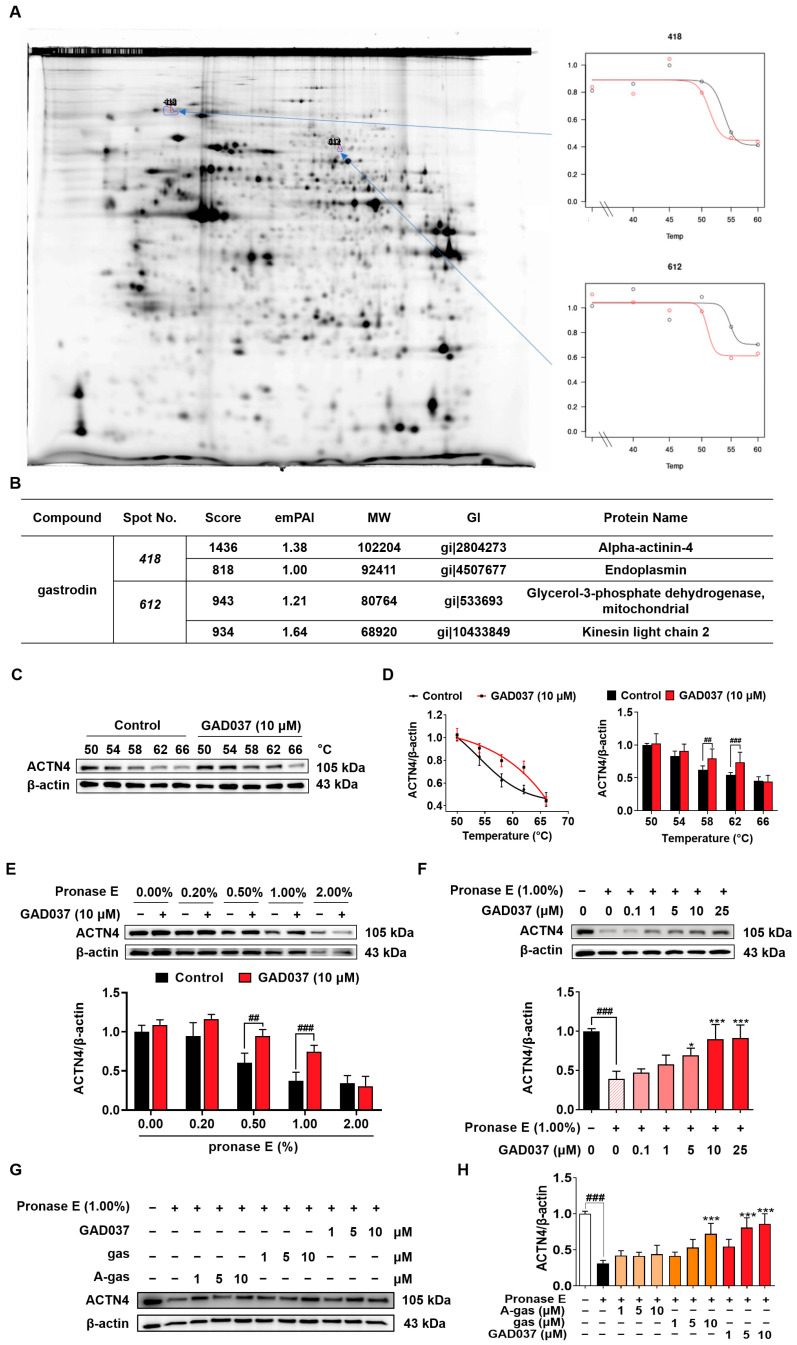
Identification of ACTN4 as a target of GAD037. (**A**) The results of 2DE-CETSA following treatment with gas at various temperatures in HeLa cells. (**B**) The LC-MS/MS analysis results for each spot in (**A**). (**C**) The Western blot analysis and (**D**) digitalized results of ACTN4 protein at varying temperatures following treatment with GAD037 at 10 µM for 4 h. (**E**) The PC12 cell lysates and GAD037 at 10 µM were incubated for 3 h at room temperature and digested with pronase E of different concentrations for 25 min, and ACTN4 protein in the lysates was determined by Western blot analysis. (**F**) The PC12 cell lysates and different concentrations of GAD037 were incubated for 3 h at room temperature and digested with pronase E (0 or 1:100 dilution) for 25 min, and ACTN4 protein in the lysates was determined by Western blot analysis. The black, light pink, pink, red represent the control, pronase E, pronase E + GAD037 (0.1, 1, 5 µM), pronase E + GAD037 (10, 25 µM) groups, respectively. (**G**) The Western blot analysis and (**H**) digitalized results of the effects of different concentrations of A-gas, gas, and GAD037 on the protein stability of ACTN4 proteins in PC12 cells. In the Western blot experiment, three dishes were also set for each group, and the extracted proteins were mixed and sampled. The data are presented as the mean ± SEM. * *p* < 0.05 and *** *p* < 0.001 represent significant differences compared with the control group; ^##^ and ^###^ indicate significant differences at *p* < 0.01 and *p* < 0.001, respectively.

During our investigation into the targets of gas, we identified that ACTN4 was a target using a 2DE-CETSA assay (Figure 5A,B). To validate whether GAD037 specifically targets ACTN4, a series of experiments, including CETSA and DARTS, were conducted. In the CETSA assay, ACTN4 experienced a shift in thermal stability following treatment with GAD037 (Figure 5C,D). Additionally, the DARTS analysis revealed that GAD037 at a concentration of 10 μM significantly inhibited the degradation of ACTN4 protein, particularly at a ratio of 1.0% (Figure 5E). As illustrated in Figure 5F, the stability of the ACTN4 protein increased in a concentration-dependent manner after treatment with GAD037, indicating that ACTN4 is indeed a potential target for GAD037. Moreover, we explored whether GAD037 enhanced the NGF-mimic activity through ACTN4. As demonstrated in Figure S6, treatment with ACTN4 siRNA reduced the percentage of cells displaying neurite outgrowth induced by GAD037 from 62.7% ± 1.8% to 33.7% ± 1.9%, suggesting a crucial role for ACTN4 in the NGF-mimic activity of GAD037. Additionally, acetyl-gastrodin (A-gas) and gas are both marketed drugs, and we compared the affinities of them with GAD037 for ACTN4 (Figure 5G,H). In the DARTS assay, GAD037 was found to enhance the stability of ACTN4 at lower concentrations compared to both A-gas and gas, indicating that GAD037 may exhibit a higher affinity for ACTN4 than these two compounds.

### 3.6. INSR and ACTN4 Play a Synergistic Role in Promoting NGF-Mimic Activity and Contribute to Neuroprotection of GAD037

**Figure 6:**
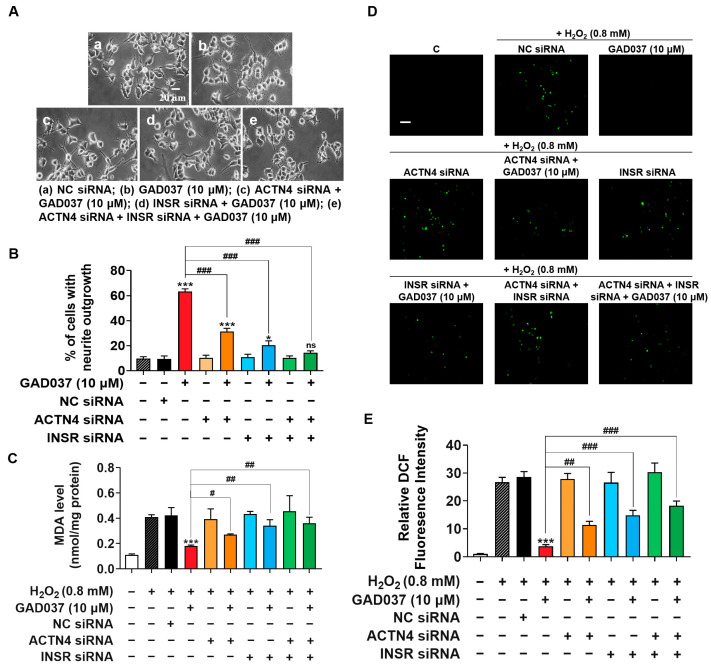
INSR and ACTN4 exhibit synergistic effects in promoting NGF-mimic activity and play pivotal role in neuroprotective activities of GAD037. (**A**) Morphological changes of neurite outgrowth in PC12 cells. (**B**) Percentage of neurite outgrowth treated with or without GAD037 for 48 h following NC siRNA, ACTN4 siRNA, INSR siRNA transfection alone, or both siRNA co-transfection in PC12 cells. (**C**) Levels of MDA content treated with or without GAD037 for 24 h following NC siRNA, ACTN4 siRNA, INSR siRNA transfection alone, or both siRNA co-transfection in H_2_O_2_-induced damage cells. (**D**) Levels of ROS treated with or without GAD037 for 24 h following NC siRNA, ACTN4 siRNA, INSR siRNA transfection alone, or both siRNA co-transfection in H_2_O_2_-induced damage cells. Microphotograph and (**E**) fluorescence intensity quantification of PC12 cells stained with DCFH-DA to detect ROS under fluorescence microscopy. Scale bar, 100 µm. After siRNA treatment, measurements of NGF-mimic activity and ROS assay were also performed in 24-well plates with two repeat wells for each group of samples. Data are presented as mean ± SEM. * *p* < 0.05 and *** *p* < 0.001 represent significant differences compared with NC siRNA group; ns indicates no significant differences compared with NC siRNA group; ^#^, ^##^, and ^###^ indicate significant differences at *p* < 0.05, *p* < 0.01, and *p* < 0.001, respectively.

To investigate the potential synergistic effects of INSR and ACTN4, their individual or combined knockdown was performed on PC12 cells. The NGF-mimic activity induced by GAD037 was assessed in cells either individually or co-transfected with INSR and ACTN4 siRNAs. As shown in Figure 6A,B, treatment with ACTN4 siRNA reduced GAD037-induced neurite growth from 63.3% ± 1.2% to 31.3% ± 1.5%, while the percentage of cells with neurite outgrowth decreased to 20.3% ± 2.0% following INSR siRNA treatment. Notably, NGF-mimic activity was further diminished after co-transfection of both siRNAs, with only 14.3% ± 0.9% of cells showing neurite outgrowth, a result not significantly different from the negative control group. These findings suggest that INSR and ACTN4 contribute to the NGF-mimic activity of GAD037 in a synergistic manner.

Furthermore, to clarify whether GAD037 exhibited neuroprotective activity through INSR and ACTN4, the knockdown of INSR and ACTN4 was implemented to assess its impact on the reduction of oxidative stress induced by GAD037. The levels of MDA and ROS were measured in PC12 cells after treatment with ACTN4 siRNA, INSR siRNA alone, or both siRNAs. As shown in Figure 6C, ACTN4 siRNA partially increased the MDA levels that were reduced by GAD037 in cells damaged by H_2_O_2_. In contrast, the use of INSR siRNA alone or co-transfected with both siRNAs significantly elevated MDA levels, which were not significantly different from those in the H_2_O_2_-treated group. This suggests that both INSR and ACTN4 are involved in the reduction of MDA induced by GAD037, with INSR playing a particularly significant role. Additionally, as illustrated in Figure 6D,E, the decrease in ROS levels induced by GAD037 was partially reversed following treatment with ACTN4 siRNA, INSR siRNA alone, or both siRNAs. This finding indicates that additional regulatory factors contribute to the reduction of ROS levels associated with GAD037. In summary, INSR and ACTN4 are critical players in the neuroprotective activity of GAD037.

To further investigate the modulation of INSR and ACTN4 by GAD037, siRNA experiments were conducted. Initially, PC12 cells were transfected with INSR siRNA to evaluate the expression of the ACTN4 protein expression. As demonstrated in Figure S7A,C, INSR siRNA did not reduce ACTN4 protein levels induced by GAD037. Furthermore, the phosphorylated INSR protein induced by GAD037 remained unchanged following the transfection of ACTN4 siRNA (Figure S7B,C). These results indicate that the regulation of INSR and ACTN4 by GAD037 is not directly related.

### 3.7. The PI3K/Akt Signaling Pathway Takes an Important Role in the NGF-Mimic Activity of GAD037

**Figure 7:**
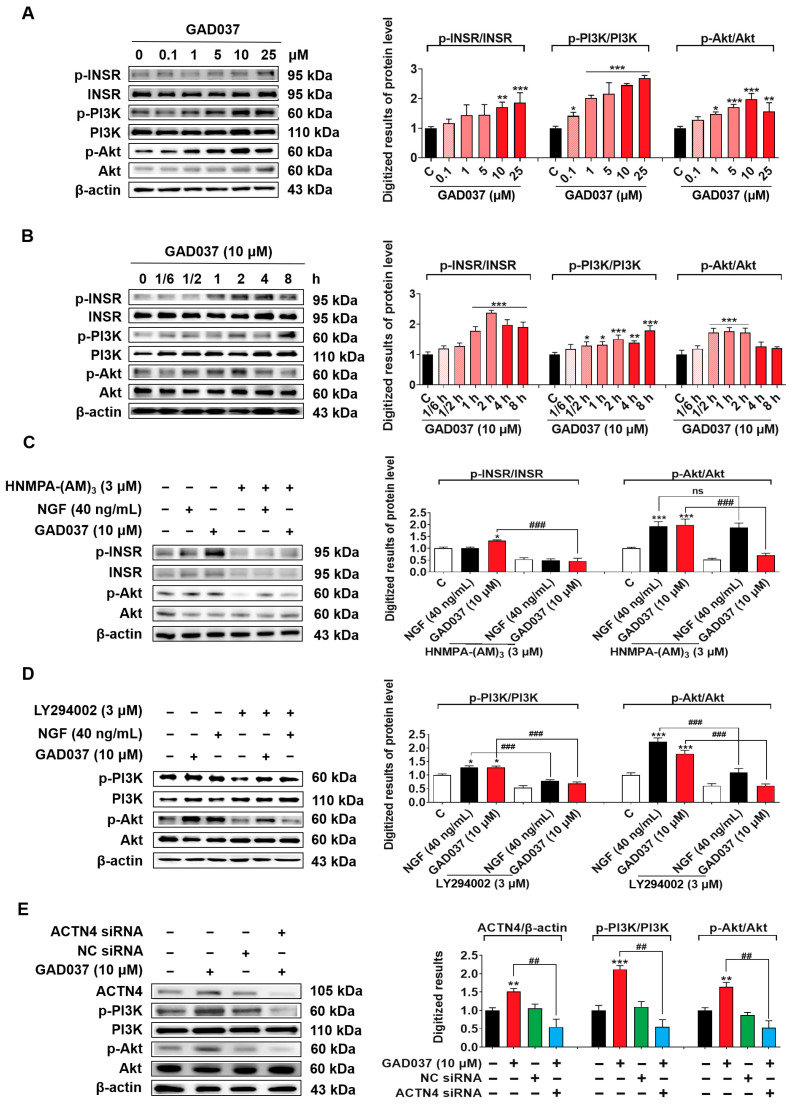
The PI3K/Akt signaling pathway is involved in the NGF-mimic activity induced by GAD037. (**A**) The Western blot analysis and digitalized results of the phosphorylation levels of INSR, PI3K, and Akt induced by GAD037 in a concentration-dependent manner (**B**) and time-dependent manner. In (**A**), the black, pink, red represent the control, GAD037 (0.1, 1, 5 µM), GAD037 (10, 25 µM) groups, respectively. In (**B**), the black, light pink, pink, red represent the control, GAD037 (1/6 h), GAD037 (1/2 h, 1 h, 2 h), GAD037 (4 h, 8 h) groups, respectively. (**C**) The Western blot analysis and digitalized results of the phosphorylation levels of INSR and Akt treated with the INSR inhibitor and subsequently GAD037 or NGF for 2 h. (**D**) The Western blot analysis and digitalized results of the phosphorylation levels of PI3K and Akt treated with PI3K inhibitor and subsequently GAD037 or NGF for 2 h. (**E**) The Western blot analysis and digitalized results of the ACTN4 protein and phosphorylated PI3K and Akt protein following transfection with either ACTN4 siRNA or NC siRNA and GAD037 treatment for 2 h. For the Western blot analysis, three dishes were used to repeat for each group, and the extracted proteins were mixed as samples. The data are presented as the mean ± SEM. * *p* < 0.05, ** *p* < 0.01, and *** *p* < 0.001 represent significant differences compared with the control group; ns indicates no significant differences; ^##^ and ^###^ indicate significant differences at *p* < 0.01 and *p* < 0.001, respectively.

The PI3K/Akt signaling pathway, identified as a common downstream mediator of INSR and ACTN4, has been reported in previous studies [12,27]. Results from PI3K inhibitor (LY294002) experiments suggest that PI3K may contribute to the NGF-mimic activity induced by GAD037 (Figure 3I). Therefore, the relationship between the PI3K/Akt signaling pathway and the proteins INSR and ACTN4 was investigated after treatment with GAD037. Initially, a concentration-response investigation was conducted to assess the phosphorylation levels of INSR, PI3K, and Akt following treatment with GAD037 at concentrations of 0.1, 1, 5, 10, or 25 µM. As illustrated in Figure 7A, GAD037 concentration-dependently increased the phosphorylation levels of these proteins. GAD037 at 10 μM is the optimal concentration. Subsequently, the time-dependent effects of GAD037 at 10 µM on phosphorylation of INSR, PI3K, and Akt over an 8 h period were assessed in Figure 7B. GAD037 enhanced INSR phosphorylation, which peaked at 2 h, PI3K phosphorylation peaked at 8 h, and Akt phosphorylation increased within 30 min and reached its peak at 1 h. Furthermore, the interactions between INSR, PI3K, and Akt were analyzed using specific inhibitors for INSR and PI3K, HNMPA-(AM)_3_ and LY294002, respectively. Notably, the INSR inhibitor substantially reduced phosphorylation of both the INSR and the Akt induced by GAD037 (Figure 7C). Similarly, the PI3K inhibitor reversed the increases in phosphorylation of PI3K and Akt instigated by GAD037 (Figure 7D). Additionally, siRNA experiments were conducted to determine whether activation of ACTN4 by GAD037 led to the regulation of the PI3K/Akt signaling pathway. Transfection with ACTN4 siRNA resulted in a notable reduction of GAD037-induced ACTN4, phosphorylation-PI3K, and phosphorylation-Akt (Figure 7E). Overall, these findings suggest that GAD037 produced the NGF-mimic effect on PC12 cells by targeting INSR and ACTN4 to activate the PI3K/Akt signaling pathway.

### 3.8. The Ras/Raf/MEK/ERK Signaling Pathway Contributed to the NGF-Mimic Activity of GAD037

**Figure 8:**
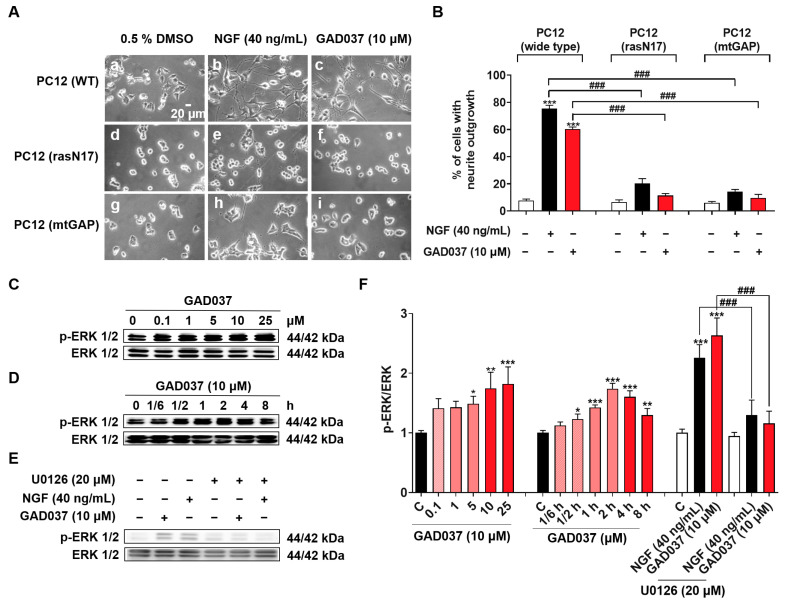
Ras/Raf/MEK/ERK signaling pathway is involved in NGF-mimic activity of GAD037. (**A**) Morphological changes of PC12 cells after treatment with GAD037 or NGF in wide-type or Ras mutant PC12 cells: (**a**) control (0.5% DMSO) in wide-type; (**b**) NGF (40 ng/mL) in wide-type; (**c**) GAD037 (10 µM) in wide-type; (**d**) control (0.5% DMSO) in Ras mutant PC12 cells (rasN17); (**e**) NGF (40 ng/mL) in Ras mutant PC12 cells (rasN17); (**f**) GAD037 (10 µM) in Ras mutant PC12 cells (rasN17); (**g**) control (0.5% DMSO) in Ras mutant PC12 cells (mtGAP); (**h**) NGF (40 ng/mL) in Ras mutant PC12 cells (mtGAP); (**i**) GAD037 (10 µM) in Ras mutant PC12 cells (mtGAP). (**B**) Percentage of neurite outgrowth induced by GAD037 or NGF for 48 h in wide-type or Ras mutant PC12 cells. (**C**) Western blot analysis for phosphorylation levels of ERK induced by GAD037 in concentration-dependent manner (**D**) and time-dependent manner. (**E**) Western blot analysis for ERK phosphorylation treated with MEK inhibitor and subsequently GAD037 or NGF for 2 h. (**F**) Digitalized results of (**C**–**E**). In (**F**), the black, pink, red represent the control, GAD037 (0.1, 1, 5 µM), GAD037 (10, 25 µM) groups, respectively. During Western blot experiment, three dishes were also set for each group, and extracted proteins were mixed as samples. Data are presented as mean ± SEM. * *p* < 0.05, ** *p* < 0.01, and *** *p* < 0.001 represent significant differences compared with control group; ^###^ indicates significant differences at *p* < 0.001.

The Ras/Raf/MEK/ERK signaling pathway has been linked to the NGF-mimic activity of GAD037, as demonstrated through experiments involving specific inhibitors (Figure 3C–E). To investigate whether the Ras/Raf/MEK/ERK signaling pathway is involved in the NGF-mimic activity of GAD037, the PC12 cell line with the Ras mutation, which included the membrane-targeted PC12 (mtGAP) or the dominant inhibitory mutant PC12 (rasN17), and a Western blot analysis were used. As expected, GAD037 failed to induce the neurite outgrowth of PC12 cells with the Ras mutant in Figure 8A,B. Furthermore, GAD037 enhanced ERK phosphorylation in a concentration-dependent manner in Figure 8C,F. ERK phosphorylation was increased at 0.5 h and arrived at a peak at 2 h after treatment with GAD037 at 10 µM (Figure 8D,F). Additionally, ERK phosphorylation in the GAD037-treated group was diminished by the MEK inhibitor, U0126, in Figure 8E,F. These findings indicate that the Ras/Raf/MEK/ERK signaling pathway plays a critical role in the NGF-mimic activity of GAD037.

## 4. Discussion

AD, a heterogeneous condition characterized by complex pathobiology, poses a significant global health challenge [1]. TCM serves as a valuable source of compounds with therapeutic potential. *Gastrodia elata* Blume is a TCM commonly employed in China for the treatment of headaches, dizziness, and strokes [16]. Gas, one of the predominant compounds found in *Gastrodia elata* Blume, exhibits antioxidant, anti-inflammatory, and neuroprotective properties [28]. Previous studies have indicated that gas plays a neuroprotective role in ameliorating AD-related pathology. However, its effective dosage in mice is a minimum of 90 mg/kg, which is less than ideal [19]. Recent studies have shown that the introduction of fluorine atoms such as those is flurithromycin can improve liposolubility and prolong the half-life of drugs [29], while modification of the glycosyl-6-ethoxyl group such as in remogliflozin etabonate confers glucosidase resistance and can improve stability [30]. To identify novel small molecules with anti-AD efficacy, we modified the chemical structure of gas by altering the substituent at the 4-position, introducing fluorine atoms to the benzene ring, and modifying the glycosyl group, resulting in the synthesis of forty derivatives. The changes in the activity and toxicity of GAD037 in Figure 1 reveal that it is a lead compound with novel NGF-mimic activity and no toxicity.

Neuronal oxidative stress promotes α-synuclein aggregation and the formation of A*β* deposits [31]. Moreover, A*β* deposits can mediate oxidative stress, thereby forming a vicious cycle [32]. Antioxidants such as resveratrol and curcumin have been reported to ameliorate cognitive function by inhibiting oxidative stress and reducing A*β* deposition [33,34]. Gas has antioxidant and neuroprotective effects [35]. Accordingly, we compared the protection of gas and GAD037 on PC12 cells under oxidative stress and A*β* conditions. The results in Figure 2 indicate that GAD037 produced neuroprotection via antioxidative stress and reduction of A*β* toxicity. Its antioxidant capacity and ability to reduce A*β* toxicity were stronger than that of gas.

In the neuroprotection assay under H_2_O_2_-induced oxidative stress, we found that the death rates of PC12 cells are very high, arriving at 60% after the H_2_O_2_ treatment. It is possible that they have a metabolic effect. To avoid the shortcomings of this method, we will select another method such as the fluorescein diacetate (FDA) staining method and flow cytometry to directly detect the dead cells in the future.

Identification of the target protein of small molecules plays an important role in drug development. It can provide important evidence for safety and drug efficacy evaluation and elucidation for the mechanism of action. At first, we focused on the signaling pathways involved in NGF-mimic activity such as the Trks, Ras/Raf/MEK/ERK, INSR/PI3K/Akt, IGF-1R/PI3K/Akt, and GR/PLC/PKC signaling pathways and used the specific protein inhibitors which the proteins located in these pathways to screen them [12,20,25]. The changes in the NGF-mimic activity of PC12 cells after treatment with GAD037 and various inhibitors in Figure 3 suggest that INSR may serve as a candidate target protein for GAD037. To gather more evidence to support this conclusion, siRNA, CETSA, and DARTS analyses were conducted. The results of these analyses, presented in Figure 4 and Figure 5, indicate that INSR is indeed the target protein through which GAD037 exhibited NGF-mimic activity in PC12 cells. Intriguingly, we found that ACTN4 was not only a potential target of gas but also a target protein of GAD037. ACTN4 is directly involved in the neurite outgrowth of PC12 cells treated with GAD037. Regretfully, we did not utilize surface plasmon resonance, cryo-electron microscopy, and AD mice knocked out for INSR and ACTN4 to confirm whether these proteins are genuine drug-target proteins and to determine their binding sites. In the future, we aim to address these aspects in our research efforts.

During the DARTS experiments, we found that the Western blot bands of the control groups after treatment with 1% pronase E were quite different in different experiments. It is possible that the storage time of pronase E affected the activity of pronase E. The activity of the newly prepared enzyme solution was high. However, the activity of the digestive enzyme gradually weakened with the increase in storage time. Thus, we will use the pronase E with the same storage period to conduct DARTS experiments in future research.

In neurons, ACTN4 promotes dendrite protrusions and maintains neuronal structure [36]. In addition, INSR signaling has been shown to affect neurite outgrowth in cultured sympathetic and sensory neurons [37] and take part in maintaining intracellular redox balance by regulating glucose metabolism [38]. To clarify how INSR and ACTN4 regulate the NGF-mimic and neuroprotective activities of GAD037, we employed siRNA to knock down the expression of INSR and ACTN4, followed by an assessment of changes in the NGF-mimic activity of GAD037 as well as ROS and MDA levels post-treatment with H_2_O_2_ and GAD037. The results presented in Figure 6 reveal that INSR and ACTN4 work synergistically to enhance the NGF-mimic activity. Meanwhile, they contribute to improving cellular resistance to oxidative damage; this effect does not occur synergistically.

Both INSR and ACTN4 are involved in the insulin signaling pathway and play a role in maintaining glucose metabolism balance. In this process, the activation of INSR promotes the translocation of glucose transporter 4 (GLUT4) to the plasma membrane [39], while ACTN4 regulates the membrane translocation of GLUT4 [40]. To investigate whether there exist interactions between INSR and ACTN4, siRNA experiments were performed. The results in Figure S7 indicate that INSR and ACTN4 independently regulate the PI3K/Akt signaling pathway. We further examined the effect of GAD037 on proteins located downstream of the INSR and ACTN4 signaling pathways. The results in Figure 7 reveal that PI3K is a key protein involved in the co-regulation of INSR and ACTN4. Additionally, the Ras/Raf/MEK/ERK signaling pathway was found to play an important role in insulin signal transduction and was involved in the NGF-mimic effect [12]. Thus, we also checked whether this signaling pathway contributes to the NGF-mimic effect of GAD037 using Ras mutants and a Western blot analysis. The results in Figure 8 clarify that the Ras/Raf/MEK/ERK signaling pathway plays an important role in the NGF-mimic activity of GAD037. This compound may also apply to the treatment of diabetes and other metabolic diseases.

Recently, compounds that directly or indirectly activate INSR have been developed, and their mechanisms of action have been elucidated. Some of these compounds are in preclinical trials or have already been approved for clinical use, primarily for the treatment of diabetes and other metabolic disorders such as NPC43 and imeglimin hydrochloride [41,42]. However, if GAD037, as an INSR activator, is to be further developed as a neurogenic or neuroprotective agent, it is crucial to evaluate whether it may induce adverse effects such as hypoglycemia. Additionally, preclinical studies suggest ACTN4 activation has certain functions, such as in SARS-CoV-2 infection [43]. Nevertheless, research on the neurogenic and neuroprotective effects of ACTN4 activation remains limited and requires further clinical trials to confirm safety and efficacy.

Interestingly, the mechanism of action of GAD037 as an NGF mimetic differs from that of other compounds, such as *β*-Cyclocitral and cucurbitacin B. *β*-Cyclocitral, derived from *Lavandula angustifolia* Mill., promotes neurite outgrowth by activating the IGF-1R/PI3K/Akt and GR/PLC/PKC signaling pathways [20]. Cucurbitacin B, the principal component found in *Cucumis melon*, targets the cofilin protein and modulates the GR and TrkA pathways to induce neurite outgrowth [21]. Despite their different structural characteristics, these molecules exhibit NGF-mimic activity by regulating various associated signaling pathways. These findings suggest that combining these compounds could enhance therapeutic outcomes in AD.

In this study, we investigated the pharmacological effects and mechanisms of GAD037 at the cell level. However, safety evaluations for GAD037 regarding its effects on AD need to be conducted using animal models. At the same time, the pharmaceutical metabolism of GAD037, whether it can cross the BBB to produce anti-AD effects, and whether it can produce better drug efficacy through combination therapy are also key issues for our future research.

## 5. Conclusions

**Figure 9:**
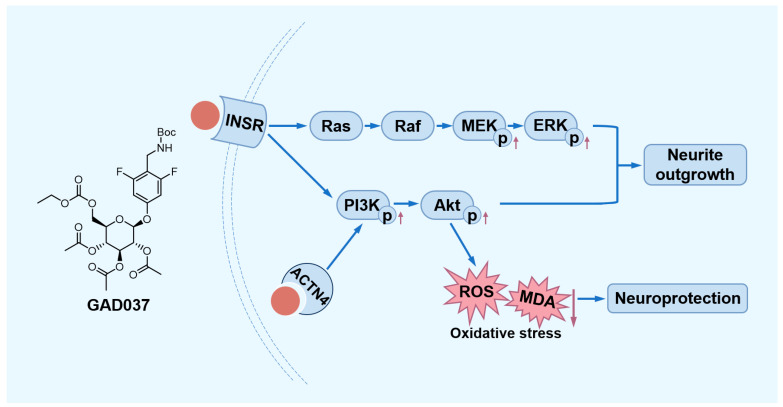
The proposed mechanism of action of GAD037. GAD037 with neuroprotection promotes neurite outgrowth by targeting INSR and ACTN4 to activate the PI3K/Akt and Ras/Raf/MEK/ERK signaling pathways. The up arrow represents an increase in the protein phosphorylation level. The down arrow represents a reduction in the ROS and MDA levels.

In summary, this study demonstrated that GAD037 exhibited exceptional NGF-mimic activity, exceeding that of gas, by targeting INSR and ACTN4 to modulate the PI3K/Akt and Ras/Raf/MEK/ERK signaling pathways in PC12 cells. Furthermore, GAD037 was shown to provide neuroprotection against oxidative stress and A*β*-induced damage (Figure 9). This research highlights the potential applications of GAD037 for its NGF-mimic and neuroprotective properties.

## Data Availability

All figures and data used to support this study are included within this article.

## Supplementary Materials

The following supporting information can be downloaded at: https://www.mdpi.com/article/10.3390/antiox14030344/s1, Supplementary Figure S1: The synthesis route and methods of GAD037; Supplementary Figure S2: The ^1^H NMR and ^13^C NMR spectrum of GAD037; Supplementary Figure S3: Determination of the optimal transfection concentration of siRNA; Supplementary Figure S4: Cell viability following exposure to gas or GAD037 at concentrations of 100 and 200 µM; Supplementary Figure S5: Morphological changes induced by H_2_O_2_ or A*β* following the treatment with GAD037 or gas in PC12 cells; Supplementary Figure S6: ACTN4 involved in NGF-mimic activity of GAD037; Supplementary Figure S7: Determination of the relationship between INSR andACTN4 induced by GAD037; Supplementary Figures S8–S13: Origin data of Western blot analysis.

## Abbreviations

The following abbreviations are used in this manuscript:

ACTN4	Alpha-actinin-4
AD	Alzheimer’s disease
A-gas	Acetyl-gastrodin
A*β*	Amyloid-*β*
CETSA	Cellular thermal shift assay
DARTS	Drug affinity responsive target stability
gas	Gastrodin
INSR	Insulin receptor
MDA	Malondialdehyde
NGF	Nerve growth factor
ROS	Reactive oxygen species
siRNA	Small interfering RNA
