# Supplementary material for: A Novel Gastrodin Derivative with Neuroprotection Promotes NGF-Mimic Activity by Targeting INSR and ACTN4 to Activate PI3K/Akt Signaling Pathway in PC12 Cells
Source: Antioxidants (Basel). 2025 Mar 14;14(3):344. doi: 10.3390/antiox14030344 (PMC11939404; doi:10.3390/antiox14030344)
Supplement: Supplementary file 1 [file antioxidants-14-00344-s001.zip › antioxidants-3474147-supplementary.pdf]

Supplementary Materials

A Novel Gastrodin Derivative with Neuroprotection Promotes NGF-Mimic Activity by Targeting INSR and ACTN4 to Activate PI3K/Akt Signaling Pathway in PC12 Cells

Jiayuan Zeng [1], Jianxia Mo [1], Makoto Muroi [2], Hiroyuki Osada [2], Lan Xiang [1,*] and Jianhua Qi [1,*]

[1]College of Pharmaceutical Sciences, Zhejiang University, Yu Hang Tang Road 866, Hangzhou 310058, China; 22219069@zju.edu.cn (J.Z.); mojx@zju.edu.cn (J.M.);

[2] Chemical Biology Research Group, RIKEN Center for Sustainable Resource Science, Wako, Saitama 351-0198, Japan; osadahiro@riken.jp (H.O.)

[*]Correspondence: lxiang@zju.edu.cn (L.X.); qijianhua@zju.edu.cn (J.Q.); Tel.: +86-571-88208627 (J.Q.)

1. Chemistry

1.1 The synthesis route and methods of GAD037

The synthesis route of GAD037 is displayed in Figure S1, and the synthesis and purification methods of GAD037 through four steps are as follows:

The synthesis and purification method of compound 1: Initially, 2,6-difluoro-4-hydroxybenzonitrile (1.00 g, 6.45 mmol, 1 equiv.) was dissolved in 1 M BH_3·THF (25.81 mL, 25.81 mmol, 4 equiv.) at 70 °C reflux. After being stirred for 6 h, the reaction mixture was quenched with 1 N HCl, adjusted the pH to 10–11 with NaOH, and extracted with ethyl acetate. The combined organic layer was dried over Na_2SO_4, filtered, and concentrated in vacuum to gain a crude product. Subsequently, the Boc protection step was performed. A solution of the crude (1.03 g, 6.45 mmol, 1 equiv.) in 1, 4-dioxane (10 mL) and H_2O (10 mL) was added with $(Boc)_2O$ (1.63 mL, 7.10 mmol, 1.1 equiv.) and 2 M Na_2CO_3 at 0 °C, kept stirring for 30 min, and then warmed up to room temperature for 30 min. The reaction mixture was terminated by the addition of ethyl acetate. The organic layer was treated as above, and then the residue obtained was purified with silica gel open column (*n*-hexane:ethyl:acetate = 20:1) to acquire a compound 1 (1.02 g, 61% yield) as a colorless solid.

The synthesis and purification method of compound 2: Compound 1 (1.02 g, 3.93 mmol, 1 equiv.) dissolved in dichloromethane (20 mL) and H_2O (20 mL) was added with tetrabutylammonium bromide (1.27 g, 3.93 mmol, 1 equiv.) and K_2CO_3 (0.98 g, 7.07 mmol, 1.8 equiv.) and the mixture was stirred at room temperature for 30 min. Subsequently, 2,3,4,6-Tetra-O-acetyl-α-D-glucopyranosyl bromide (2.10 g, 5.11 mmol, 1.3 equiv.) was added to the mixture and stirred at 50 °C for 6 h. Then the reaction mixture was extracted with CH_2Cl_2. The combined organic layer was treated as above. The crude was purified with column chromatography (*n*-hexane:ethyl acetate = 8:2) to obtain compound 2 (1.18 g, 52% yield) as a white solid.

The synthesis and purification method of compound 3: By dissolving compound 2 (1.18 g, 2.00 mmol, 1 equiv.) in the saturated methanol of $NaHCO_3$ (10 mL) equipped with a magnetic stirrer at room temperature for 1 h. The reaction mixture was

concentrated, and then added with diethylpyrocarbonate (DEPC) (0.37 mL, 2.40 mmol, 1.2 equiv.) and $Sc(OTf)_3$ (0.05 g, 0.10 mmol, 0.05 equiv.) in toluene (20 mL) and ethyl alcohol (5 mL) and kept stirring for 8 h. The reaction mixture was concentrated, and the residue obtained was purified with silica gel open column (*n*-hexane:ethyl acetate = 1:1) to gain compound 3 (0.66 g, 66% yield) as a white solid.

The synthesis and purification method of compound 4 (GAD037): A solution of compound 3 (0.91, 1.84 mmol, 1 equiv.) in pyridine (15 mL) was added with acetic anhydride (1.04 mL, 11.0 mmol, 6 equiv.) at room temperature. After being stirred for 24 h, the reaction mixture was extracted three times with ethyl acetate and 1 N HCl. The combined organic layer was treated as above. The crude was isolated with column chromatography (*n*-hexane:ethyl:acetate = 75:25) to acquire GAD037 (0.82 g, 98% yield) as a white solid.

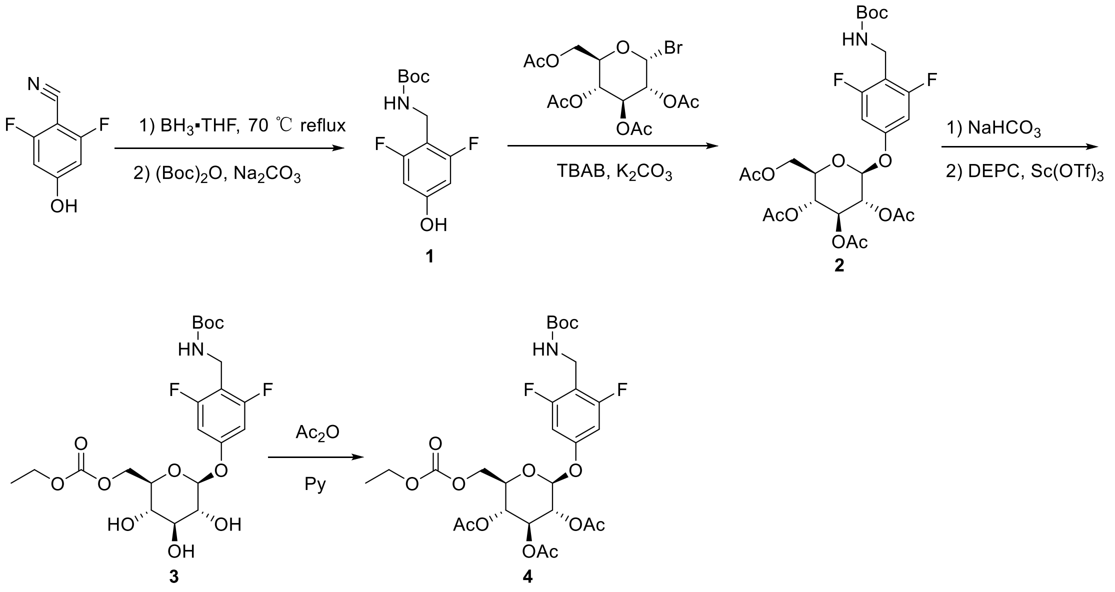

Figure S1. The synthesis route of GAD037.

1.2 ^{1}H NMR and ^{13}C NMR spectrum of GAD037

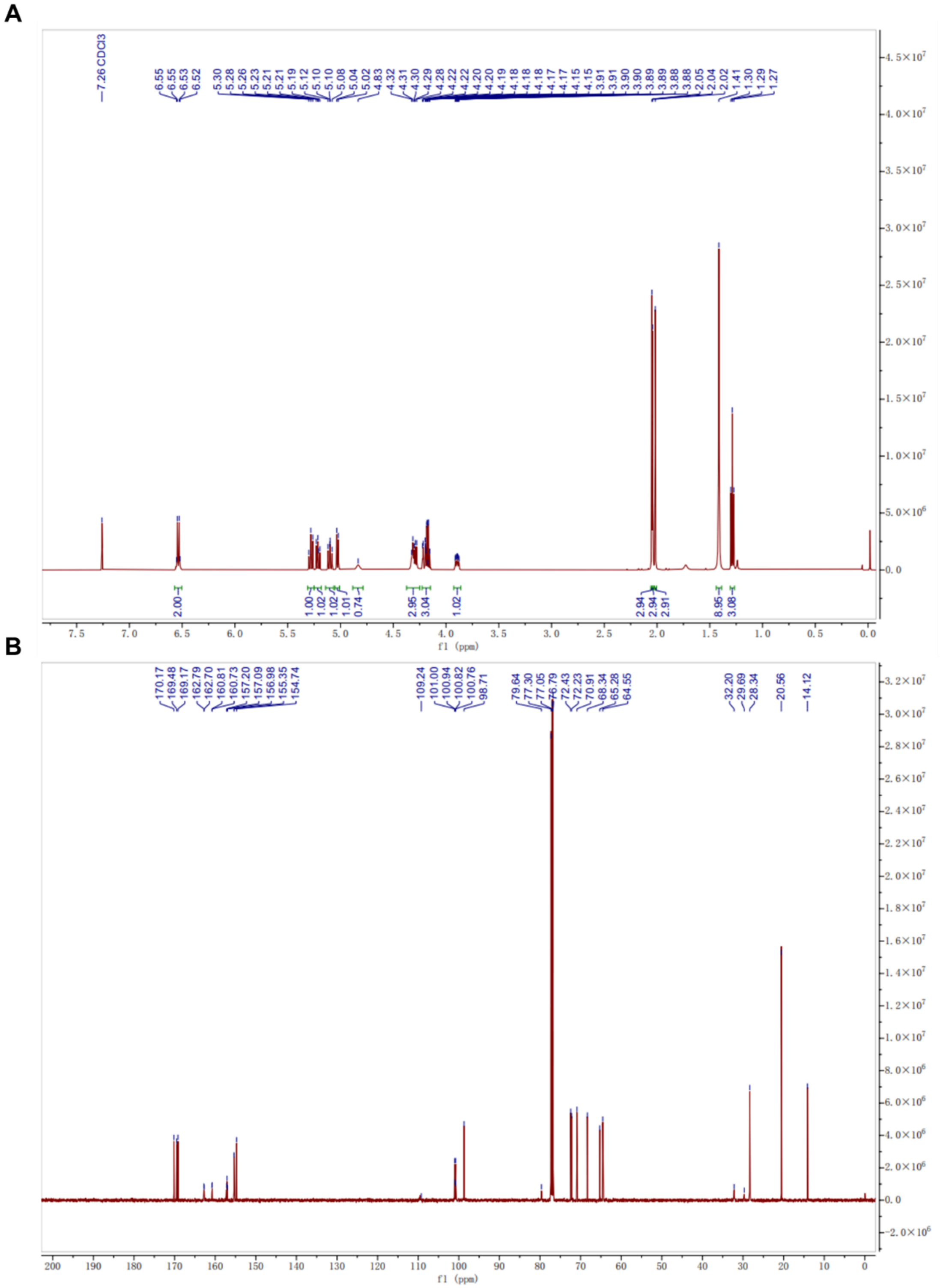

Figure S2. (**A**) ^{1}H NMR (500 MHz, $CDCl_3$) spectrum of GAD037. (**B**)^{13}C NMR (125 MHz, $CDCl_3$) spectrum of GAD037.

2. Biology

2.1 Determination of the optimal transfection concentration of siRNA.

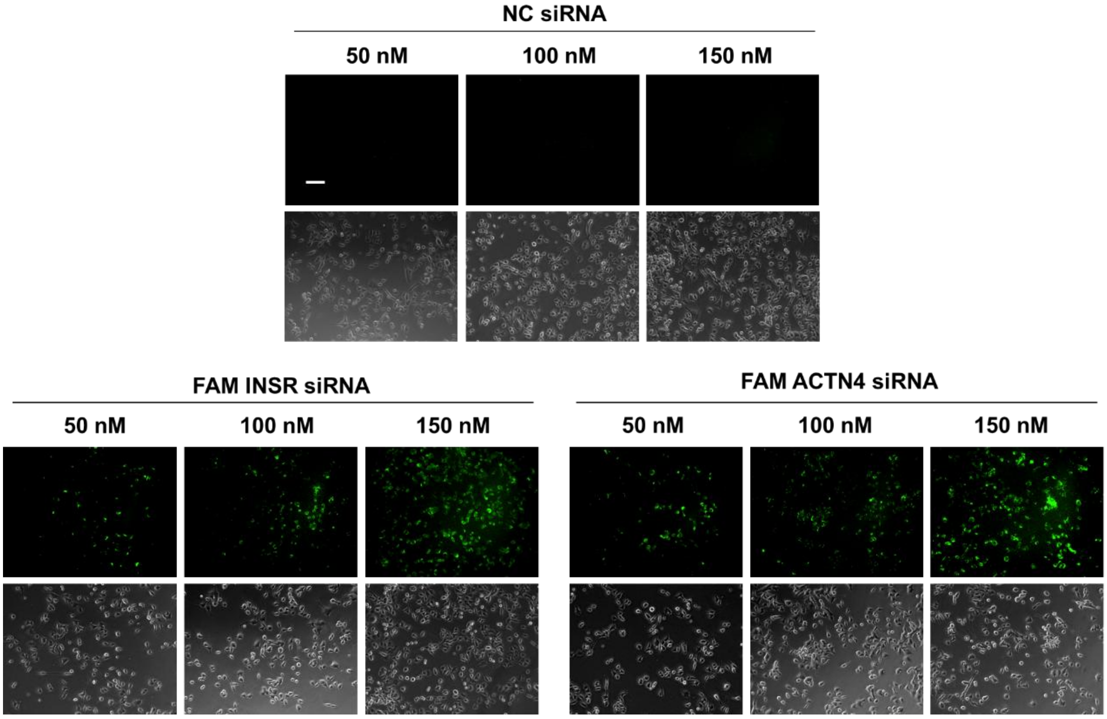

Figure S3. Microphotograph of PC12 cells after transfection with different concentrations of NC siRNA, FAM INSR siRNA, or FAM ACTN4 siRNA alone (50 nM, 100 nM, and 150 nM, respectively). The optimal concentration was chosen to achieve exceeding 90% of PC12 cells to fluoresce by observing and photographing the cells under a fluorescence microscope. Scale bar, 100 μm.

2.2 Cytotoxicity evaluation of gas and GAD037.

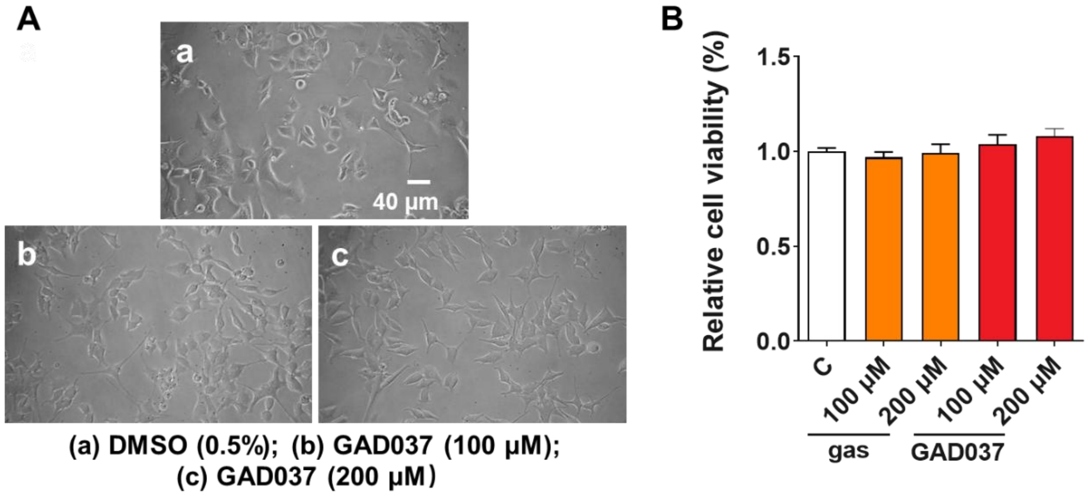

Figure S4. Effects of gas and GAD037 on cell viability in PC12 cells. (A) Morphological changes following treatment with GAD037 or gas in PC12 cells. (B) Cell viability following exposure to gas or GAD037 at concentrations of 100 and 200 μM for 24 h. Three repeats of each experiment were conducted, and the data were presented as mean ± SEM.

2.2 GAD037 improves H_2O_2 or Aβ induced morphological changes in PC12 cells

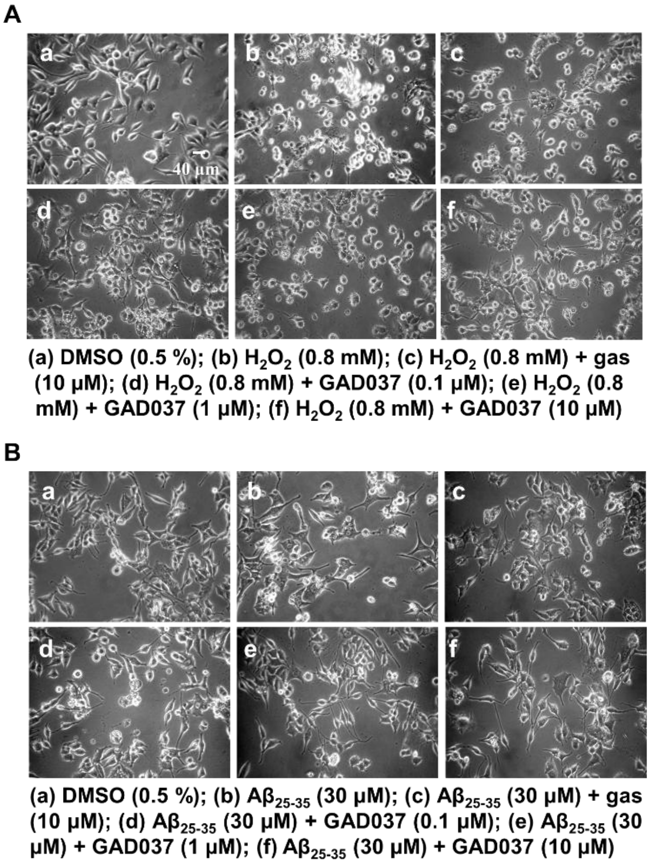

Figure S5. Morphological changes induced by H_2O_2 or A*β* following treatment with GAD037 or gas in PC12 cells. (**A**) Morphological changes in H_2O_2-induced PC12 cells after being treated with GAD037 or gas. (**B**) Morphological changes in A*β*-induced PC12 cells following the treatment with GAD037 or gas. Three repeats of each experiment were conducted.

2.3 ACTN4 contributes to NGF-mimic activity of GAD037

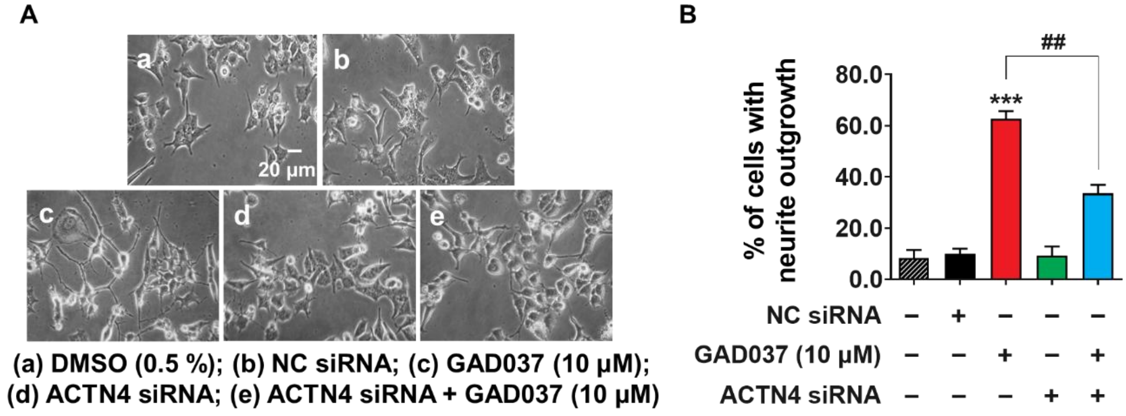

Figure S6. ACTN4 is involved in the NGF-mimic activity of GAD037. (**A**) Morphological changes of neurite outgrowth in PC12 cells. (**B**) Percentage of the neurite outgrowth treated with or without GAD037 (10 μM) following NC siRNA or ACTN4 siRNA transfection (150 nM, respectively) in PC12 cells. Three repeats of each experiment were conducted, and the data were presented as mean ± SEM. ***$p < 0.001$ represented significant differences compared with the control group; ## indicated significant differences at $p < 0.01$.

2.4 GAD037-regulated INSR and ACTN4 do not exist direct association

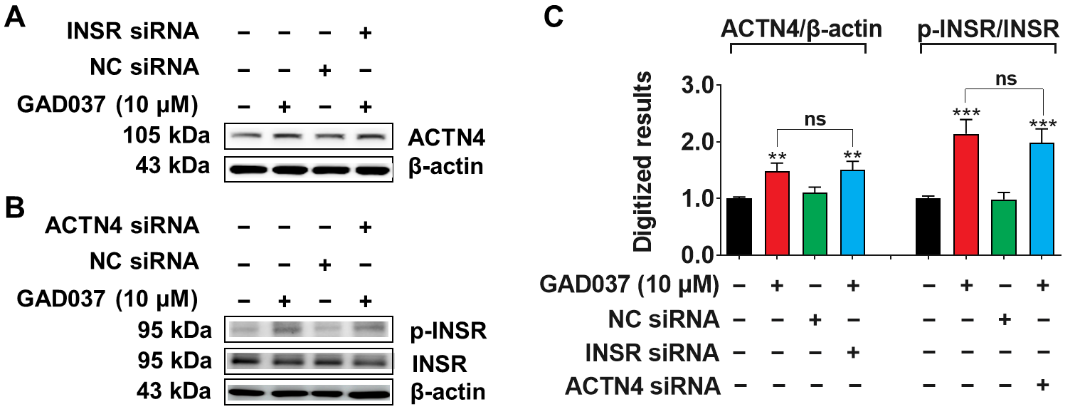

Figure S7. Determination of the relationship between INSR andACTN4 induced by GAD037. (**A**) Western blot analysis for ACTN4 protein following transfection with either INSR siRNA or NC siRNA, and GAD037 treatment. (**B**) Western blot analysis for the INSR protein and phosphorylated INSR protein after transfection with ACTN4 siRNA or NC siRNA alone, and treated with GAD037. (**C**) The digitalized results of Figures S6A,B. The data were presented as mean ± SEM. ***p* < 0.01 and ****p* < 0.001 represented significant differences compared with the control group; ns indicated no significant differences.

2.5 Origin data of Western blot analysis

The first result of Figure S8:

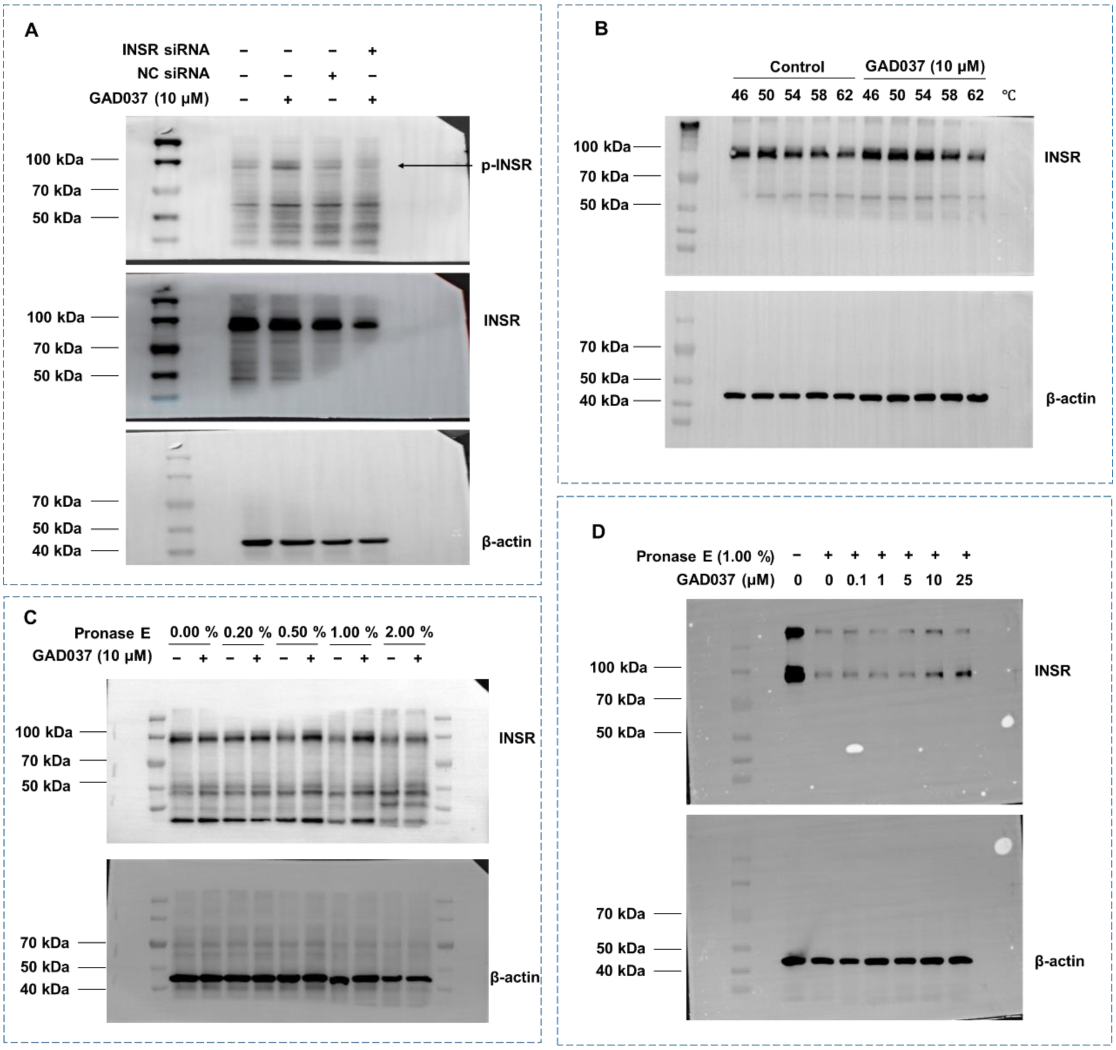

The second result of Figure S8:

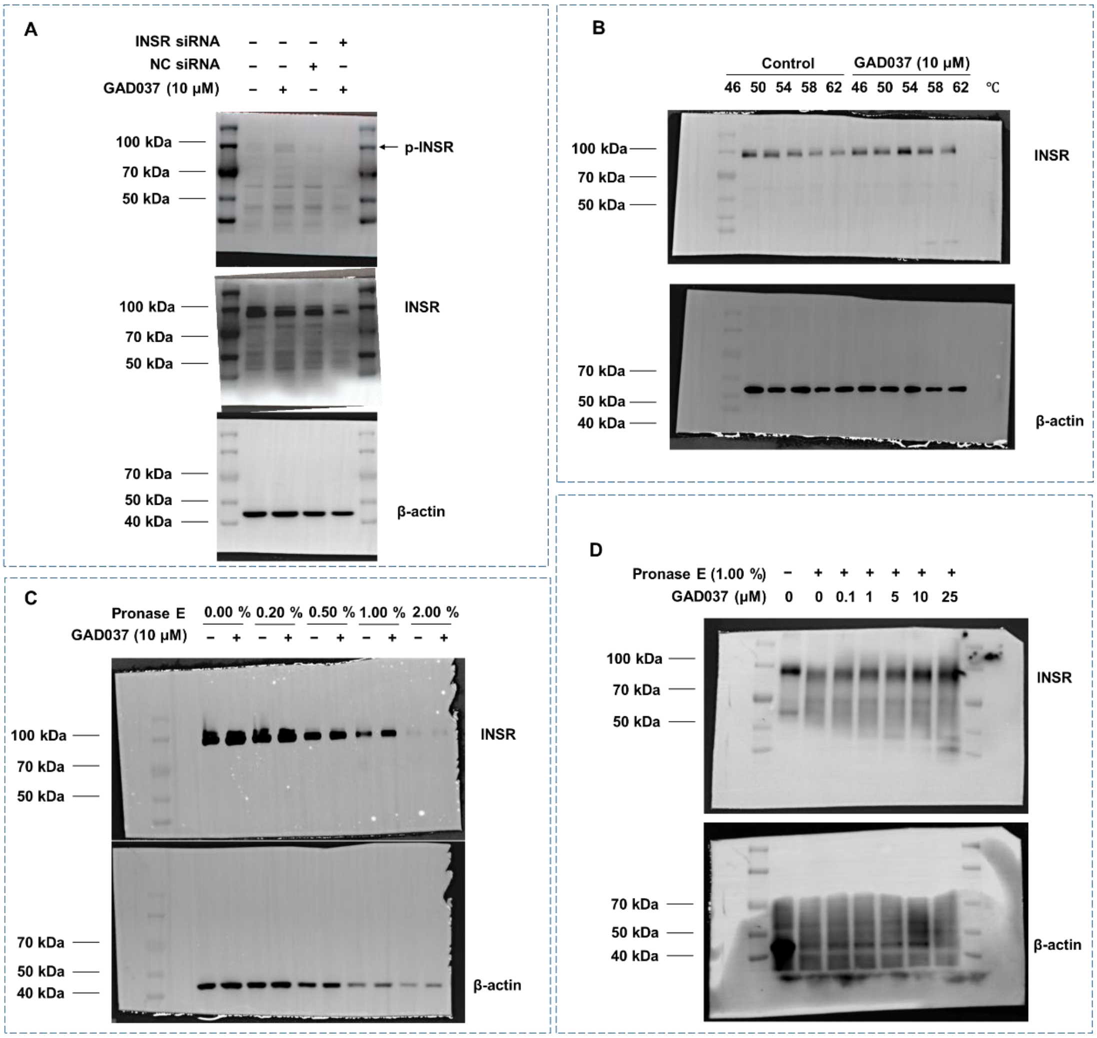

The third result of Figure S8:

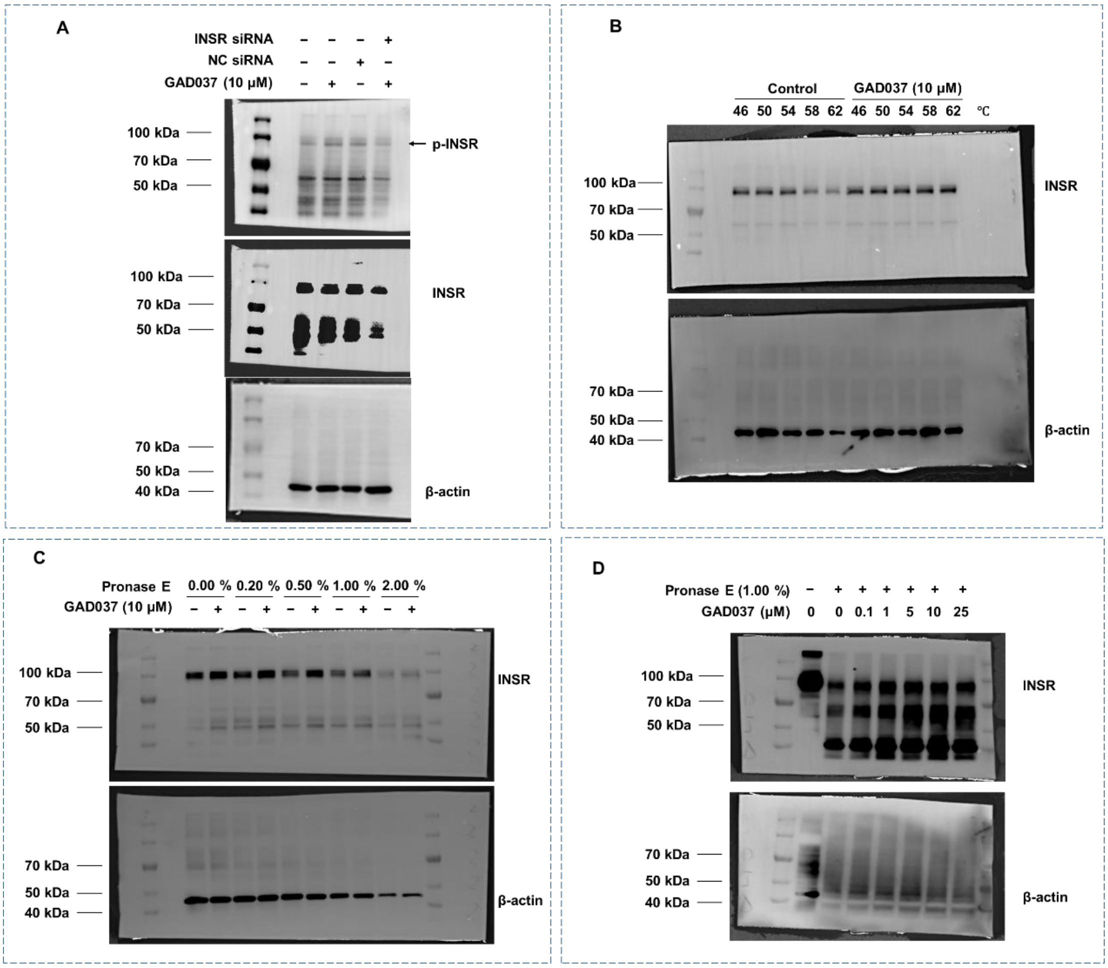

Figure S8. Original data of Western blot analysis for identification of INSR as a target of GAD037 in Figure 4. (**A**) Original data of Western blot analysis of p-INSR, INSR, and β-actin proteins following transfection with either INSR siRNA or NC siRNA, and GAD037 treatment in Figure 4A. (**B**) Original data of Western blot analysis of INSR and β-actin proteins after being treated with GAD037 at 10 μM and heating the samples at different temperatures, respectively, in Figure 4B. (**C**) Original data of Western blot analysis of INSR and β-actin proteins after being treated with GAD037 at 10 μM and digested with different concentrations of pronase E, respectively, in Figure 4C. (**D**) Original data of Western blot analysis of INSR and β-actin proteins after being treated with different concentrations of GAD037 and digested with pronase E (0 or 1.00%) in Figure 4D.

The first result of Figure S9:

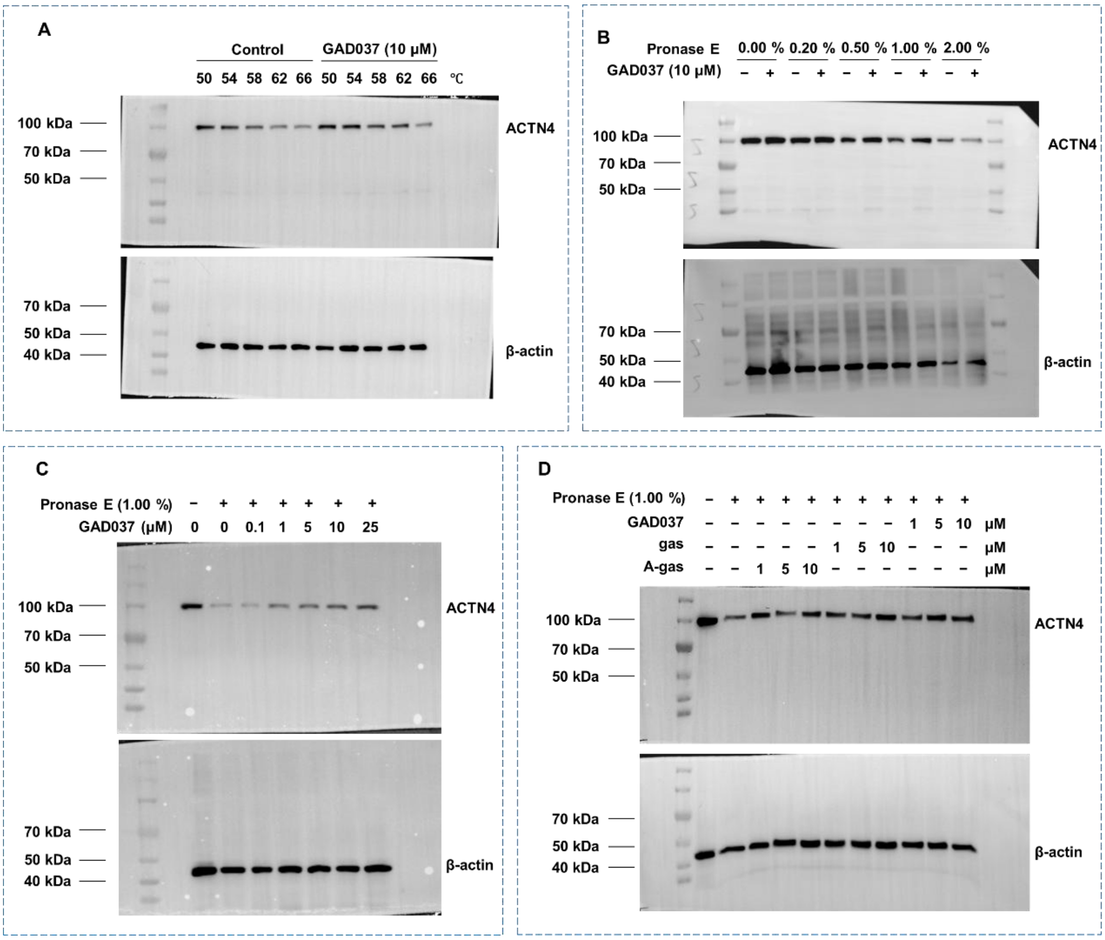

The second result of Figure S9:

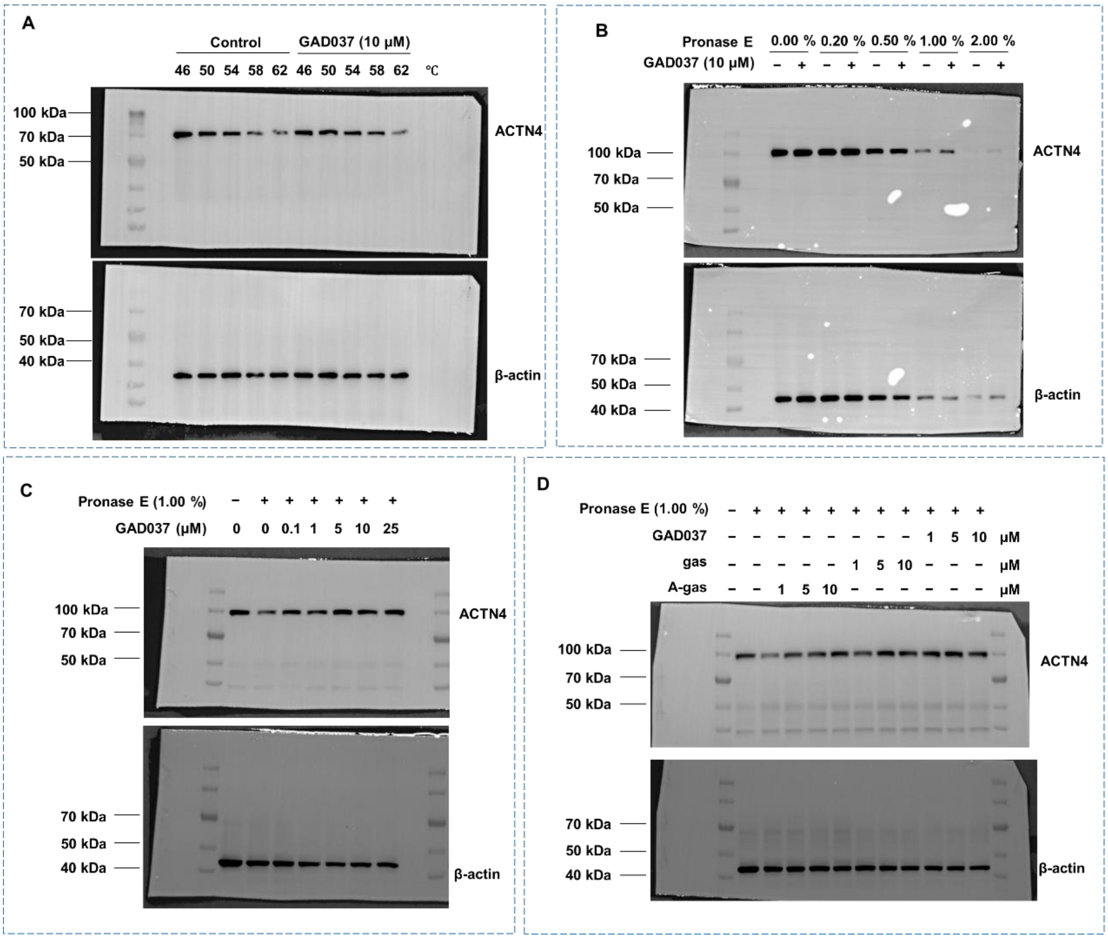

The third result of Figure S9:

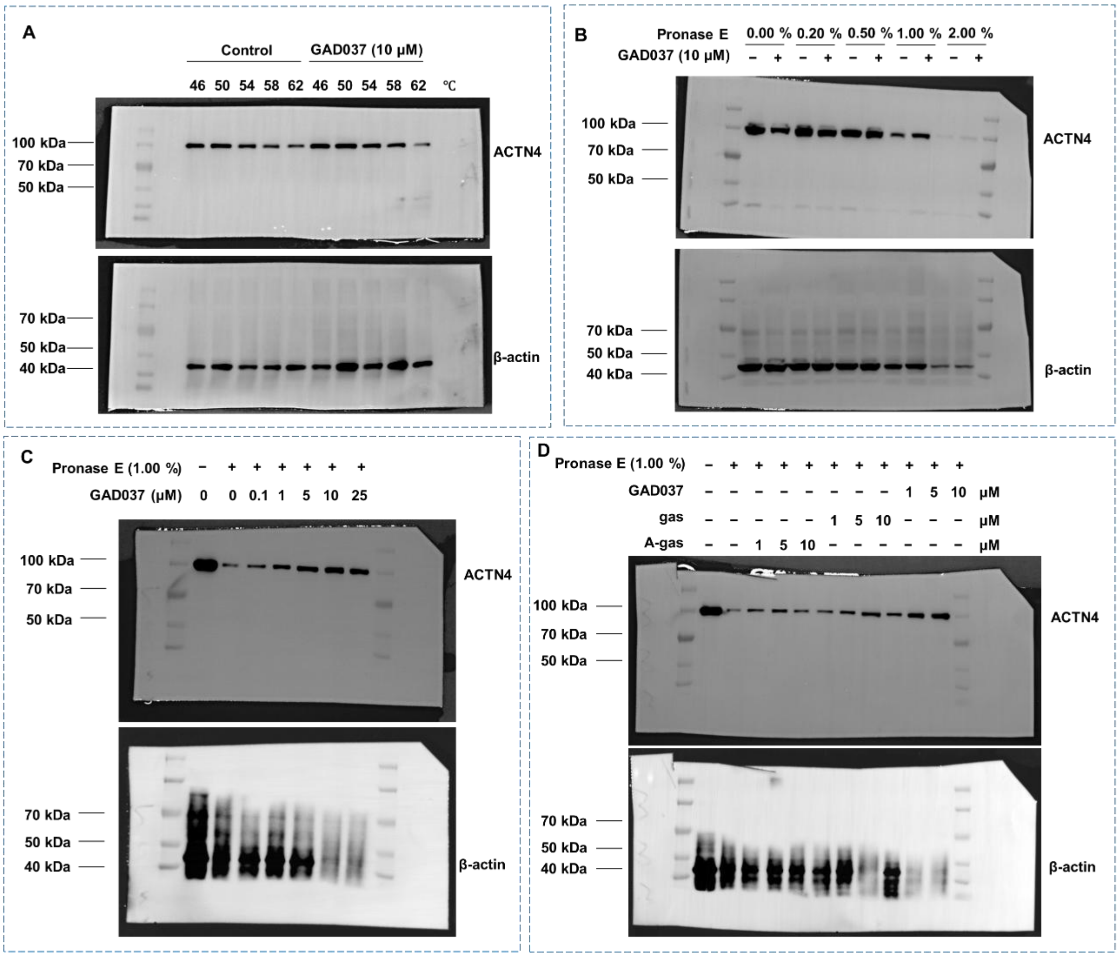

Figure S9. Original data of Western blot analysis for determination of ACTN4 as a target of GAD037 in Figure 5. (**A**) Original data of Western blot analysis of ACTN4 and β-actin proteins at varying temperatures following the treatment of GAD037 at 10 μM in Figure 5C. (**B**) Original data of Western blot analysis of ACTN4 and β-actin proteins after being treated with GAD037 at 10 μM and digested with different concentrations of pronase E, respectively, in Figure 5E. (**C**) Original data of Western blot analysis of ACTN4 and β-actin proteins after being treated with different concentrations of GAD037 and digested with pronase E (0 or 1.00%) in Figure 5F. (**D**) Original data in Figure 5G represented the effects of different concentrations of acetyl-gas, gas, and GAD037 on the protein stability of ACTN4.

The first result of Figure S10:

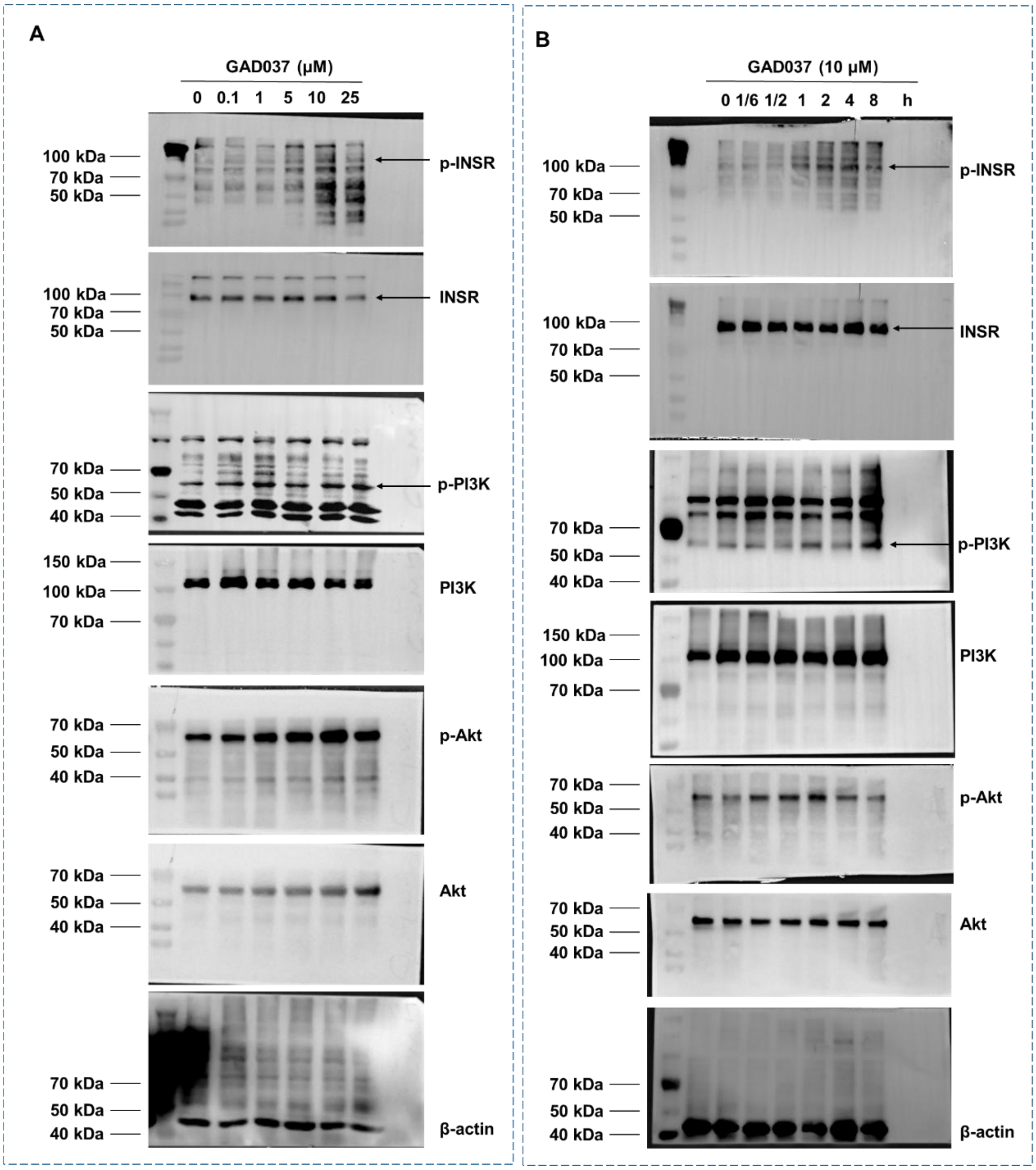

The second result of Figure S10:

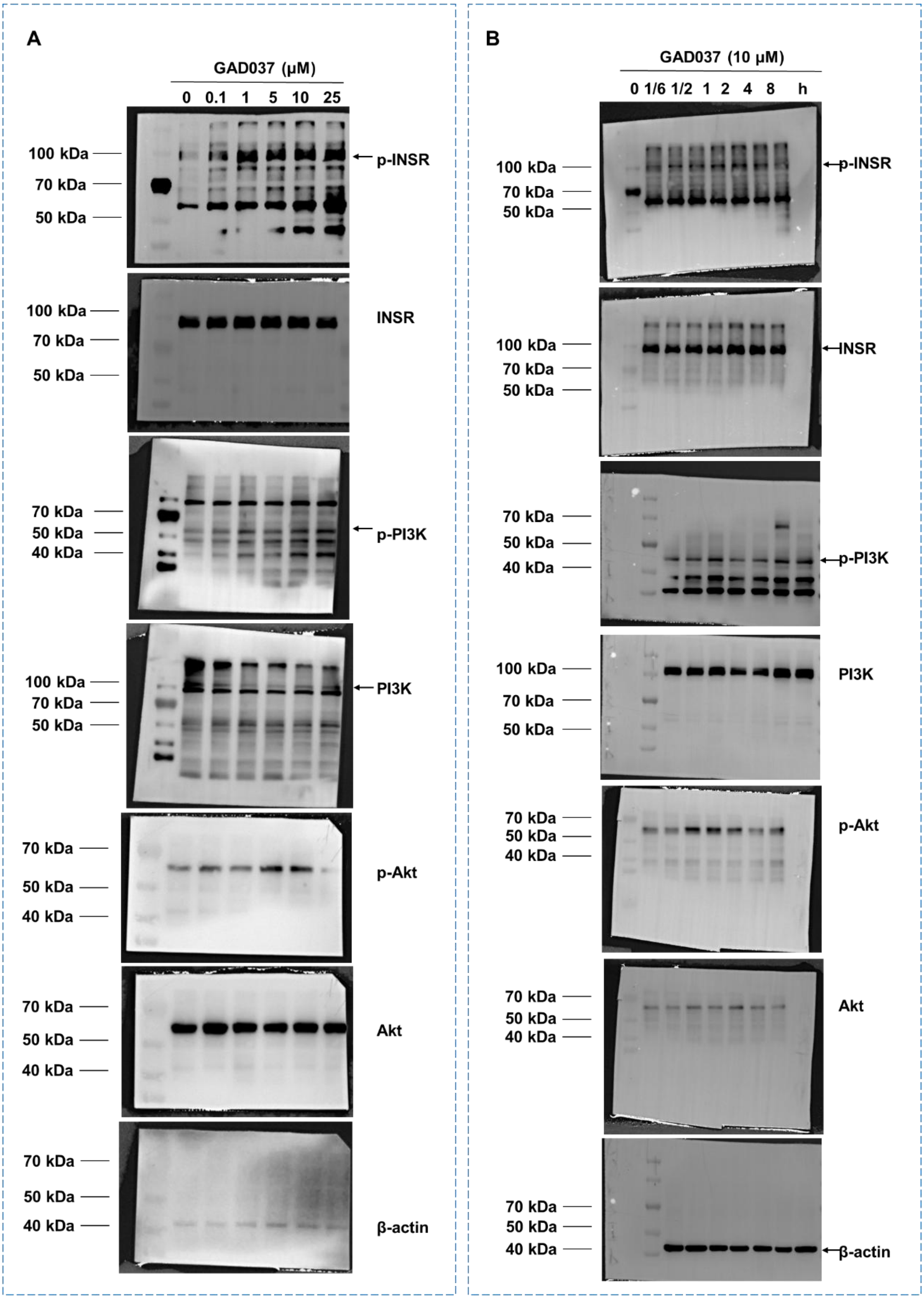

The third result of Figure S10:

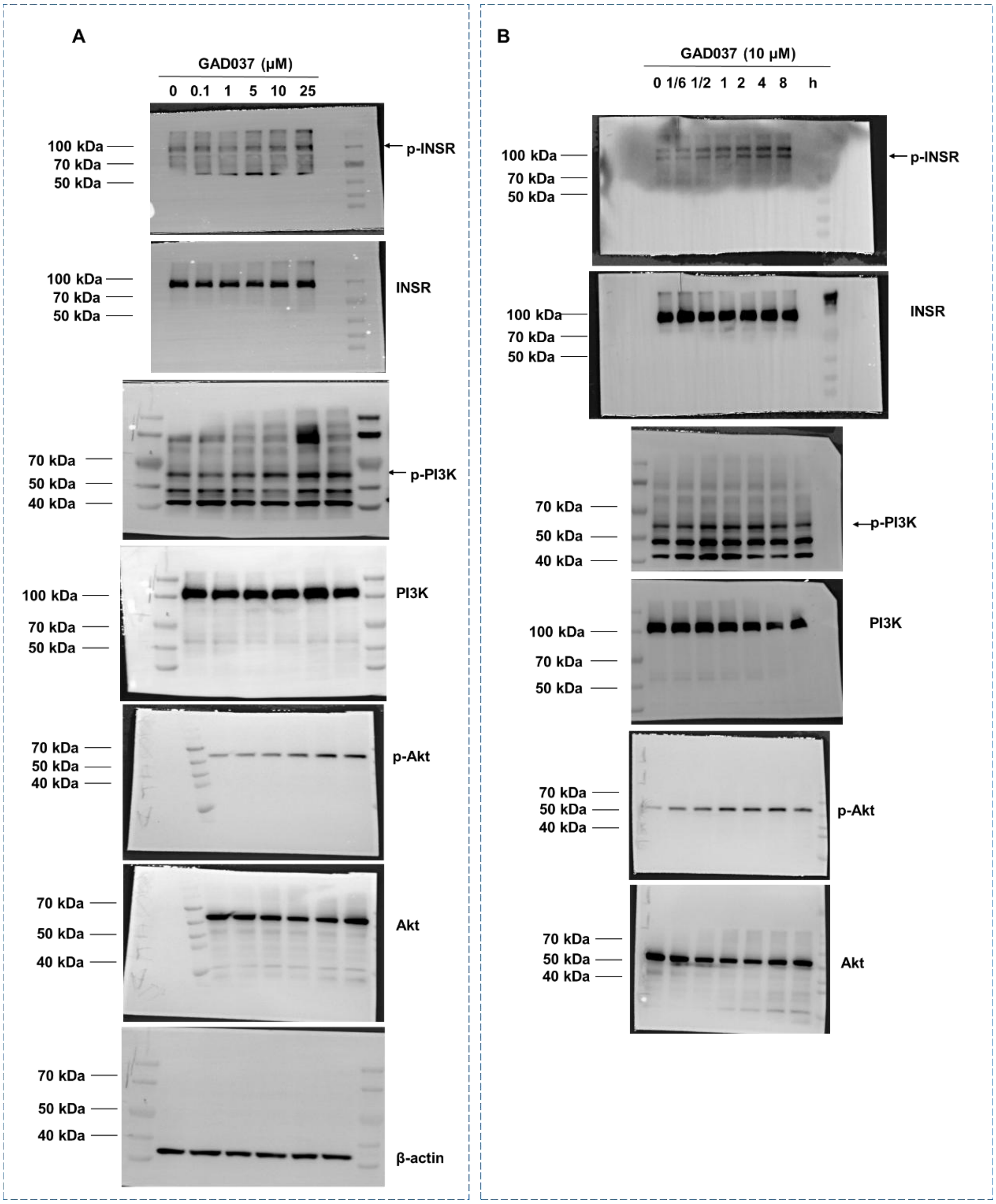

Figure S10. Original data of Western blot analysis for investigation of the dose and time relationship of INSR, PI3K, and Akt phosphorylated induced by GAD037 in Figure 7A-7B. (**A**) Original data in Figure 7A represented the phosphorylation levels of INSR, PI3K, and Akt induced by GAD037 in a dose-dependent manner. (**B**) Original data in Figure 7B represented the phosphorylation levels of INSR, PI3K, and Akt induced by GAD037 in a time-dependent manner.

The first result of Figure S11:

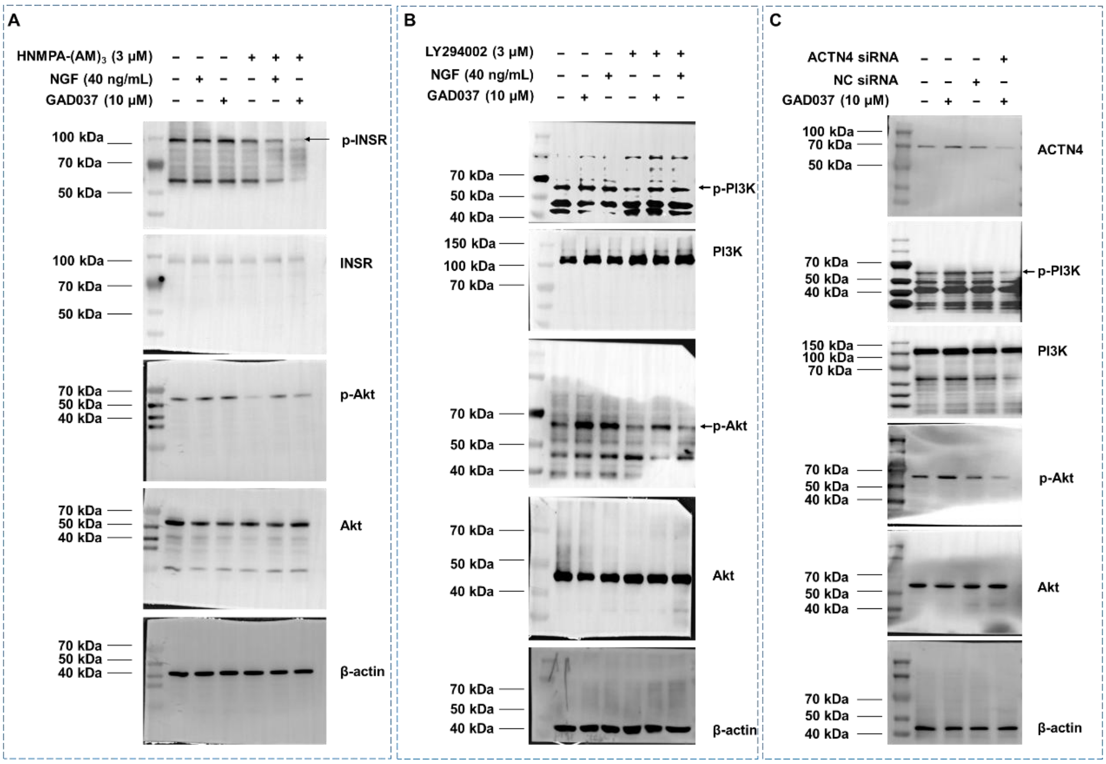

The second result of Figure S11:

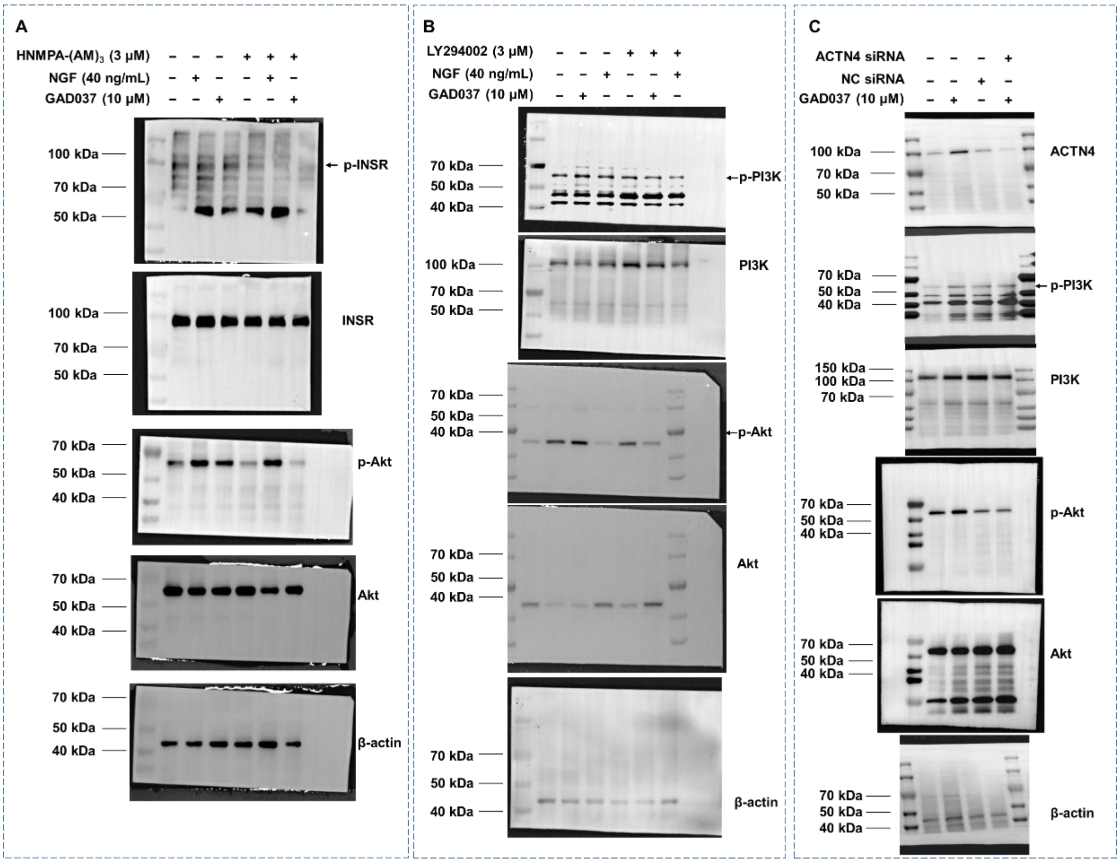

The third result of Figure S11:

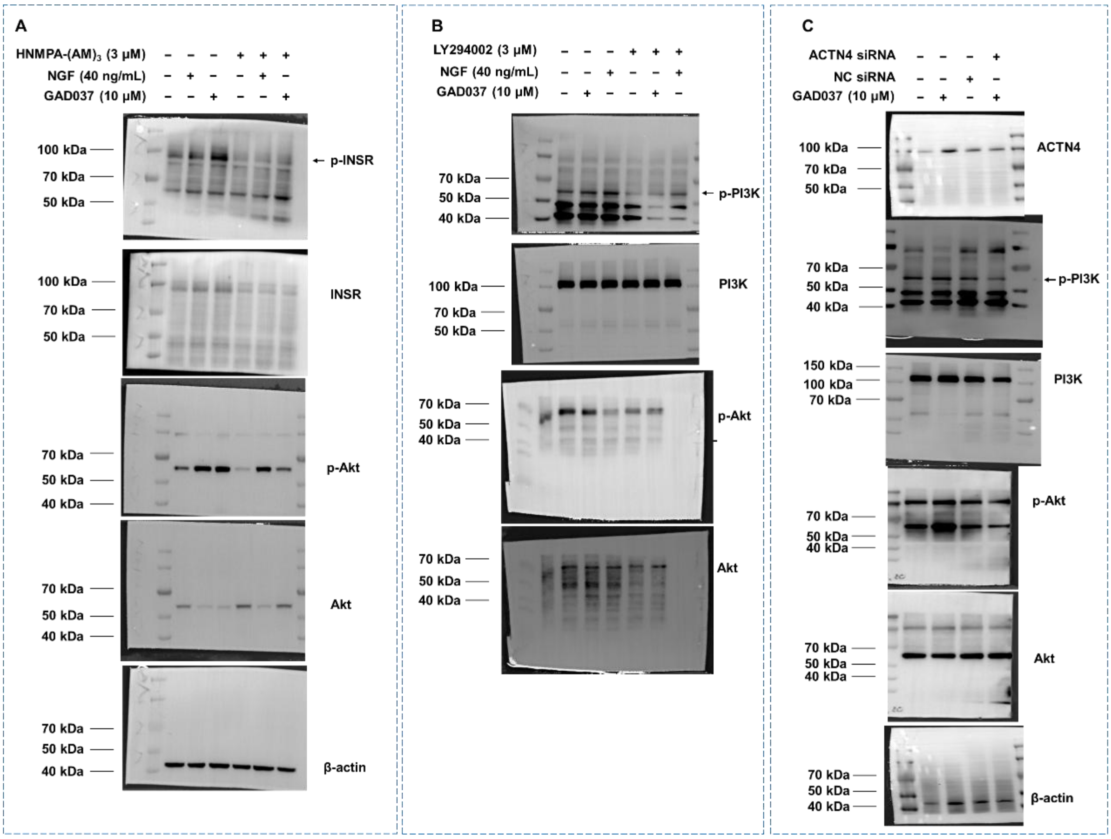

Figure S11. Original data of Western blot analysis for determination of the relationship of INSR, ACTN4, PI3K, and Akt induced by GAD037 in Figure 7C-7E. (**A**) Original data of Western blot analysis of p-INSR, INSR, p-Akt, Akt, and β-actin proteins treated with INSR inhibitor and subsequently GAD037 or NGF treatment in Figure 7C. (**B**) Original data of Western blot analysis of p-PI3K, PI3K, p-Akt, Akt, and β-actin proteins after the treatment of PI3K inhibitor and subsequently GAD037 or NGF treatment in Figure 7D. (**C**) Original data of Western blot analysis of ACTN4, p-PI3K, PI3K, p-Akt, Akt, and β-actin proteins following transfection with either ACTN4 siRNA or NC siRNA, and GAD037 treatment in Figure 7E.

The first result of Figure S12:

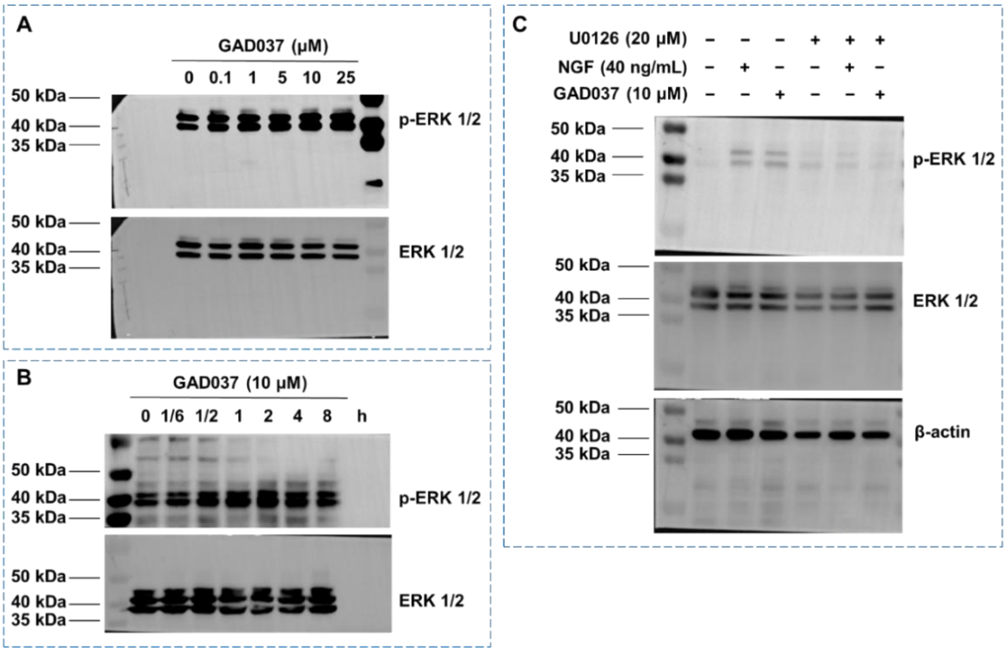

The second result of Figure S12:

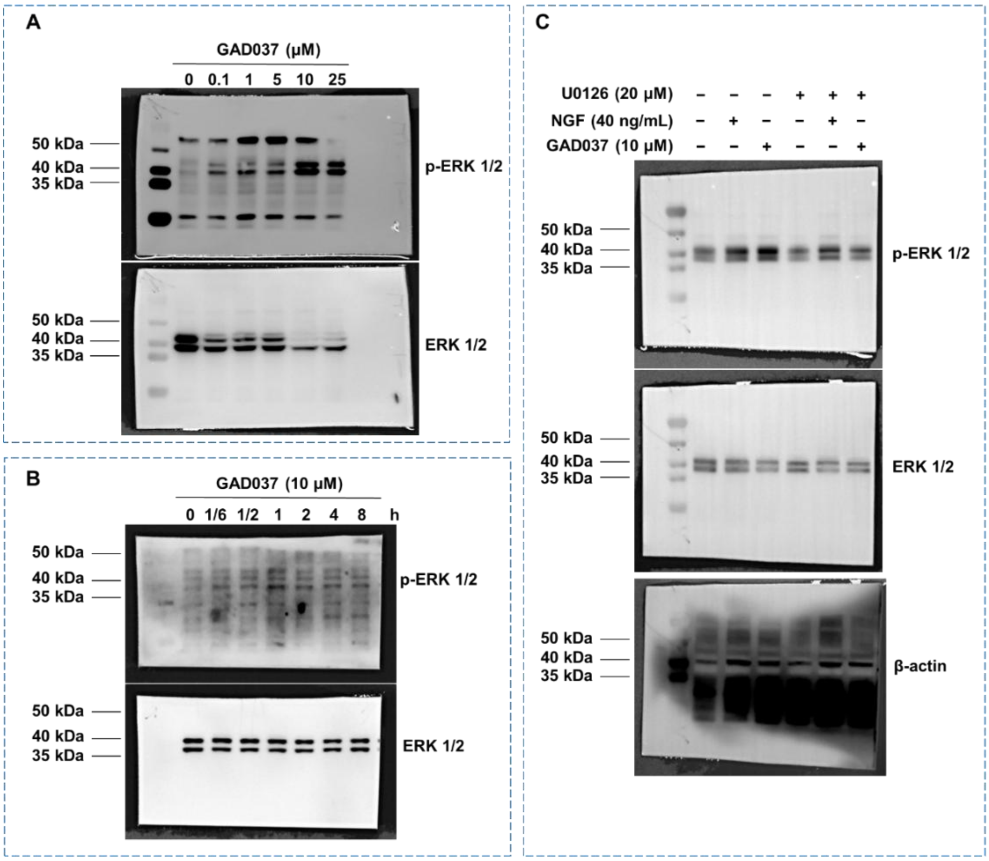

The third result of Figure S12:

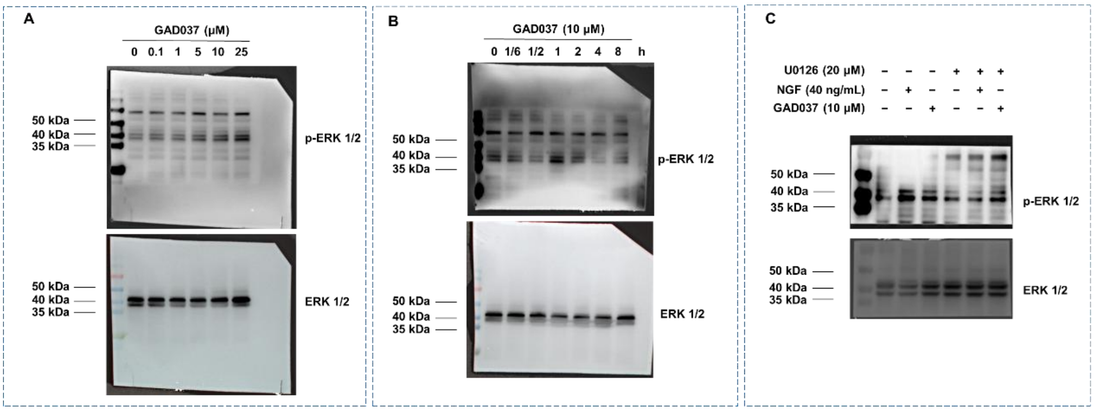

Figure S12. Original data of Western blot analysis for Ras/Raf/MEK/ERK signaling pathway related to NGF-mimic activity of GAD037 in Figure 8. (**A**) Original data of Western blot analysis of the dose relationship of ERK phosphorylated induced by GAD037 in Figure 8C. (**B**) Original data of Western blot analysis of the time relationship of ERK phosphorylated induced by GAD037 in Figure 8D. (**C**) Original data of Western blot analysis of p-ERK, ERK, and β-actin proteins treated with MEK inhibitor and subsequently GAD037 or NGF treatment in Figure 8E.

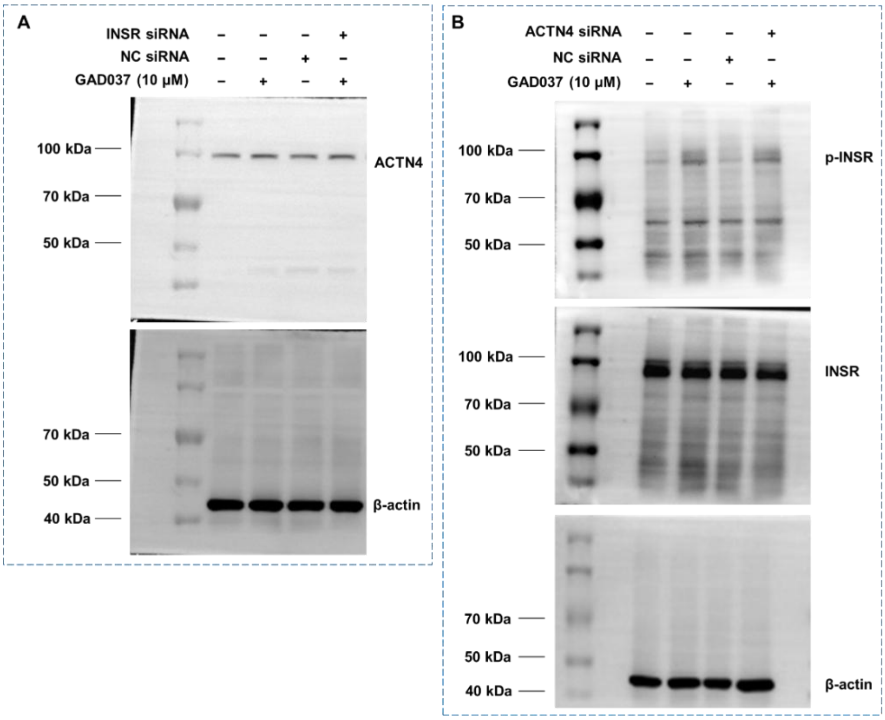

Figure S13. Original data of Western blot analysis for the relationship between INSR andACTN4 induced by GAD037 in Figure S7A-S7B. (**A**) Original data of Western blot analysis of ACTN4 and β-actin proteins following transfection with either INSR siRNA or NC siRNA, and GAD037 treatment in Figure S7A. (**B**) Original data of Western blot analysis of p-INSR, INSR, and β-actin proteins following transfection with either ACTN4 siRNA or NC siRNA, and GAD037 treatment in Figure S7B.
